# Spatiotemporal Variation Characteristics and Driving Mechanisms of Net Primary Productivity of Vegetation on Northern Slope of Tianshan Mountains Based on CASA Model, China

**DOI:** 10.3390/plants14162499

**Published:** 2025-08-12

**Authors:** Yongjun Du, Xiaolong Li, Xinlin He, Quanli Zong, Guang Yang, Fuchu Zhang

**Affiliations:** 1 College of Water Conservancy & Architectural Engineering, Shihezi University, Shihezi 832000, China; du_yongjun@stu.shzu.edu.cn (Y.D.); lixiaolong409@shzu.edu.cn (X.L.); yangguang@shzu.edu.cn (G.Y.); zfc@stu.shzu.edu.cn (F.Z.); 2 Key Laboratory of Cold and Arid Regions Eco-Hydraulic Engineering of Xinjiang Production & Construction Corps, Shihezi University, Shihezi 832000, China; 3 College of Resources and Environment, Qingdao Agricultural University, Qingdao 266109, China

**Keywords:** net primary productivity, climate change, human activities, optimal parameter-based geographic detector model, northern slope of Tianshan Mountains

## Abstract

Net primary productivity (NPP) reflects the carbon sequestration capacity of terrestrial ecosystems and it is used as an important indicator for measuring ecosystem quality. However, due to the effects of “warming and humidification” and “oasisization”, the spatiotemporal evolution and driving mechanisms of the NPP of vegetation in the northern slope of the Tianshan Mountains (NSTM), a typical arid area in China, are still unclear. Thus, in this study, we used remote sensing data and meteorological data to construct a Carnegie–Ames–Stanford–Approach (CASA) model for estimating the NPP of vegetation in the study area. Trend analysis, partial correlation analysis, and optimal parameter-based geographic detector (OPGD) methods were combined to explore the spatiotemporal evolution and driving mechanisms to changes in the NPP. The results showed that from 2001 to 2020, the annual average NPP on the NSTM exhibited an overall significant upward trend, increasing from 107.33 gC⋅m^−2^⋅yr^−1^ to 156.77 gC⋅m^−2^⋅yr^−1^, with an increase of 2.47 gC⋅m^−2^ per year and 46.06% year-on-year. Over the past 20 years, climate change and human activities generally positively affected the changes in NPP in the study area. Human activities in the study area are mainly manifested in the large-scale conversion of other land use types into farmland, with a total increase of 16,154 km^2^ in farmland area, resulting in a net increase of 6.01 TgC in NPP. Precipitation has the strongest correlation with NPP in the study area, with a partial correlation coefficient of 0.30, temperature and solar radiation have partial correlation coefficients with NPPs of 0.17 and 0.09, respectively. Therefore, increases in precipitation, temperature, and solar radiation have a promoting effect on the growth of NPP on the NSTM. During the study period, the land use type and soil moisture were the main factors that affected the spatial differentiation of vegetation NPP, and the effects of human interference on natural environmental conditions had significant impacts on vegetation NPP in the area. Therefore, in this study, we accurately determined the spatiotemporal variations in the NPP on the NSTM and comprehensively explored the driving mechanisms to provide a theoretical basis for sustainable development in arid areas and achieving carbon neutrality goals.

## 1. Introduction

Terrestrial ecosystems are critical for the global carbon cycle, converting atmospheric CO_2_ into organic compounds [1]. Vegetation is an important component of terrestrial ecosystems and it provides abundant natural resources for human survival, as well as serving as a link in material cycles and energy flows between the soil and atmosphere, thereby playing significant roles in regulating the terrestrial carbon balance and maintaining global climate stability [2,3]. Net primary productivity (NPP) is the total amount of organic matter produced by photosynthesis per unit area of vegetation per unit time, and the organic mass that remains after autotrophic respiration by vegetation [4,5]. The NPP can reflect the carbon sequestration capacity of vegetation and it is used as an important indicator for measuring the quality of an ecosystem [6,7]. Understanding NPP is critical for assessing the productivity of vegetation, carbon sequestration capacity, and overall health of terrestrial ecosystems [8,9]. The northern slope of the Tianshan Mountains (NSTM) are located in an arid area of China with a simple ecosystem structure, which is vulnerable to climate change and human activities [10]. Due to advances in science, technology, and social economic development, many grasslands and xerophytic shrub deserts on the NSTM have been developed into farmland, resulting in the accelerated evolution and rapid expansion of oases since the 1950s [11,12]. In addition, Xinjiang is located in the arid region of northwest China, where the trend of the “warming and humidification” of the climate is becoming increasingly obvious [13]. The temperature rise in the region is faster than that of other arid areas [14], thereby inevitably speeding up the water cycle, accelerating the melting of glaciers and snow in the alpine belt, changing the form of runoff supply, and increasing the uncertainty of mountain runoff to reduce regional water reserves and affect the habitat distribution pattern of vegetation [15,16]. Therefore, in the context of ongoing climate change and human activities, it is necessary to comprehensively explore the spatiotemporal evolution of NPP on the NSTM and the driving mechanisms in order to provide a scientific reference for regulating the carbon cycle in ecosystems and achieving the “dual carbon” goals.

Accurately calculating the regional NPP is the basis for studying its spatiotemporal changes [17]. The traditional NPP estimation methods are mainly based on field measurements, which are generally divided into two parts: the aboveground biomass and underground biomass. In this method, NPP data are collected within the selected ecological type range and the “point to grid cell” method is used to extrapolate these data to the regional scale or a larger scale [18]. In addition, the NPP determination method varies for different vegetation types [19]. The traditional method achieves relatively high accuracy in measuring the NPP of vegetation, but it consumes large amounts of manpower and material resources; it is also relatively difficult to study long-sequence NPP using this method and extrapolating the spatial scale has some limitations [20]. Due to research into vegetation NPP and the development of remote sensing technology, the model building method has been widely used to study the vegetation NPP at different times and in different regions. These methods include mechanistic models, such as the BIOME–BGC model [21], TEM model [22], and BEPS model [23]; statistical models, such as the Miami model [24] and Chikugo model [25]; and parametric models, such as the LUE–VPM model [26], EC–LUE model [27], and CASA model [27]. In addition, deep neural network models based on NDVI, land cover, meteorological, and DEM data have been used for NPP estimation in recent years [28]. In particular, the CASA model based on light use efficiency (LUE) data has a solid theoretical foundation and it is one of the most widely used NPP estimation models throughout the world. The CASA model can be adjusted for different ecosystems and climate types, as well as for regional or global NPP estimation [29,30]. For example, Potter et al. [31] estimated forest NPP in Southeast Asia based on the CASA model combined with MODIS remote sensing data. In some studies, high-resolution Landsat land cover data were integrated into an improved CASA ecosystem model, thereby enhancing the accuracy of NPP estimation for terrestrial ecosystems in the continental United States [32]. Furthermore, the CASA model has been continuously improved to enhance the accuracy of simulations. In particular, Zhu et al. [33] adjusted the maximum light energy utilization rate parameter in the model based on NPP data measured in China, making it more suitable for estimating the NPP of vegetation in China. Many studies have used the CASA model to investigate the NPP of vegetation in different watersheds and regions, thereby demonstrating that human activities and climate change jointly affect the temporal and spatial evolution of NPP, but the extents of their effects vary significantly among different regions, depending mainly on the implementation of local policies, ecological projects, urbanization processes, and other projects [7,34,35]. In previous studies, the CASA model was used to couple climate models and land use models to predict NPP under different future scenarios in order to determine a suitable path for future development in the study region [30].

Studying the driving mechanisms to changes in NPP is highly significant for the regulation of NPP and it has become a research hotspot in recent years [10,36,37]. Climate change and human activities are two important factors that affect changes in vegetation NPP. Climate can affect NPP directly or indirectly through precipitation, temperature, solar radiation, and other climatic factors that regulate community biomass (e.g., length of growing season, vegetation metabolism, and stomatal behavior) [38,39,40]. The relationships between the NPP of vegetation and precipitation, temperature, and radiation vary among different geographical locations and climate zones. In addition, the effects of these three factors on vegetation interact with each other. Some studies have shown that grassland NPP is more sensitive to temperature in China’s alpine region on the Qinghai–Tibet Plateau [41,42]. In Southwest China, solar radiation is the main factor related to increases in farmland NPP [43], whereas precipitation has a restrictive effect on increases in vegetation NPP [44]. In arid areas, precipitation has a major effect on the changes in NPP because precipitation can replenish soil moisture, alleviate water stress, and increase stomatal conductance, thereby increasing the absorption of CO_2_ by vegetation [45,46]. Land use changes are the most direct manifestation of the interaction between human activities and the natural environment, and they have important impacts on the stability of ecosystems [10,20,23]. Land use changes have two main impacts on NPP, where the implementation of ecological engineering can promote increases in the vegetation NPP [7], whereas the accelerated development of urbanization leads to decreases in NPP [10].

The NSTM is located in the arid region of northwest China. In recent years, the “warming and humidification” trend has intensified in this region, where population and urbanization issues have become more prominent since the implementation of the “Belt and Road” initiative and the overall plan for the Xinjiang Free Trade Pilot Zone in recent years. Developing artificial oases has become an important strategic goal in the arid region of Xinjiang [47,48]. The area of artificial oases on the NSTM has been increasing continuously for a long time, and the degree of “oasification” has risen continuously [10,48]. However, the driving mechanism to the effects of climate and human activities on NPP remain unclear. In addition, the relationships between vegetation dynamics and environmental change are complex, and most previous studies used correlation and residual trend analysis to quantitatively assess the impacts of environmental change on vegetation dynamics [35,49,50]. These methods all assume that significant linear relationships exist between driving factors and changes in vegetation [51]. However, the relationships between vegetation and driving factors are nonlinear, and affected by a variety of complex environmental factors [52]. Therefore, considering the spatiotemporal heterogeneity between driving factors related to vegetation is crucial for accurately assessing their nonlinearity and volatility. In order to address the shortcomings of previous research, we quantified the impacts of climate and human activities on changes in NPP due to the intensification of climate “warming and humidification” and “oasification” on the NSTM. In addition, we employed the optimal parameter-based geographic detector (OPGD) model to quantitatively assess the power of changes in driving factors for explaining changes in vegetation NPP. Compared with the traditional geographic detector model (GDM), the OPGD model can avoid the subjective effects of the spatial scale and data discretization processing, and identify the optimal combination of the spatial scale, spatial data discretization method, and number of partitions [53]. The OPGD method can explore the interaction between two factors from a spatiotemporal heterogeneity perspective to evaluate whether the combination of two factors will increase or decrease the power of a single related factor for explaining changes in NPP, thereby obtaining deeper insights into the interaction between the changes in NPP and driving factors [54,55].

Therefore, this study takes the NSTM as the study area. Based on the estimation results of vegetation NPP in the region from 2001 to 2020, it analyzes the spatiotemporal variation characteristics of NPP, quantifies the impact of climate change and human activities on NPP, and explores the driving mechanism of NPP change. The main research objectives are (1) to estimate the NPP on the NSTM from 2001 to 2020 and analyze its spatiotemporal distribution characteristics and changing patterns; (2) to quantify the interannual impact of human activities and climate change on NPP based on the changing trends of human activities and climate change; and (3) to clarify the driving mechanism of NPP changes from the perspective of spatiotemporal heterogeneity. As the core area of the “Belt and Road” economic zone, clarifying the relationship between the changing characteristics of ecosystem NPP and its driving factors can provide a scientific basis for the region’s environmental management and sustainable development.

## 2. Results

### 2.1. Temporal and Spatial Variations in NPP

**Figure 1:**
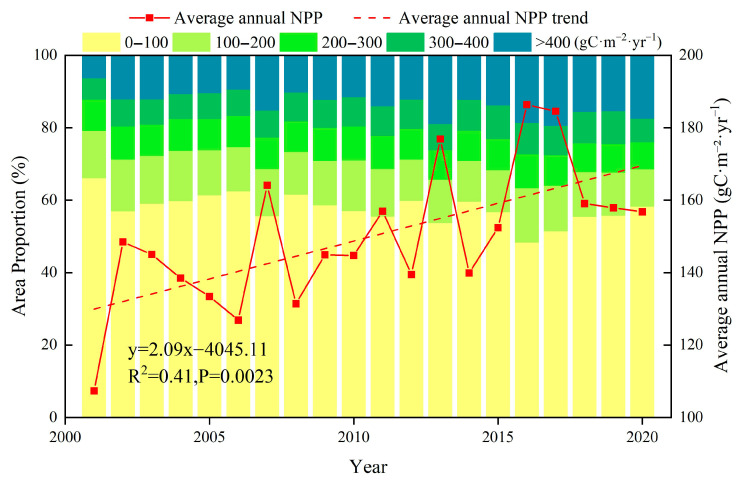
Variation in average annual NPP and the proportion of different grades of NPP.

Figure 1 shows the annual average NPP changes in the study area and the proportion of different grades of NPP, the annual average NPP on the NSTM ranged from 107.33 to 186.38 gC⋅m^−2^⋅yr^−1^ from 2001 to 2020, with a generally significant upward trend, and an increase of 2.47 gC⋅m^−2^ per year (R^2^ = 0.41, *p* = 0.0023) and 46.06% year-on-year. The multi-year average NPP and total NPP were 149.75 gC⋅m^−2^⋅yr^−1^ and 43.49 TgC (1 Tg = 1 × 10^12^ g), respectively, and the maximum NPP of 186.38 gC⋅m^−2^⋅yr^−1^ occurred in 2016, which was higher than the average by 36.63 gC⋅m^−2^⋅yr^−1^. The minimum NPP of 107.33 gC⋅m^−2^⋅yr^−1^ occurred in 2001, and it was lower than the average by 42.42 gC⋅m^−2^⋅yr^−1^. In addition, we divided the NPP of the NSTM from 2001 to 2020 into five grades: 0–100, 100–200, 200–300, 300–400, and >400 gC⋅m^−2^⋅yr^−1^, and we statistically analyzed the area proportion of each grade in different years. The statistical results show that the changes in the trends among different NPP grades were significantly different. In many years, the NPP < 100 gC⋅m^−2^⋅yr^−1^ interval accounted for the largest proportion, but it tended to fluctuate downward, decreasing from 66.17% to 58.30% after 20 years, whereas the proportion in the NPP > 400 gC⋅m^−2^⋅yr^−1^ interval increased from 6.29% to 17.43%, which was basically consistent with the changing trend in the average annual NPP.

**Figure 2:**
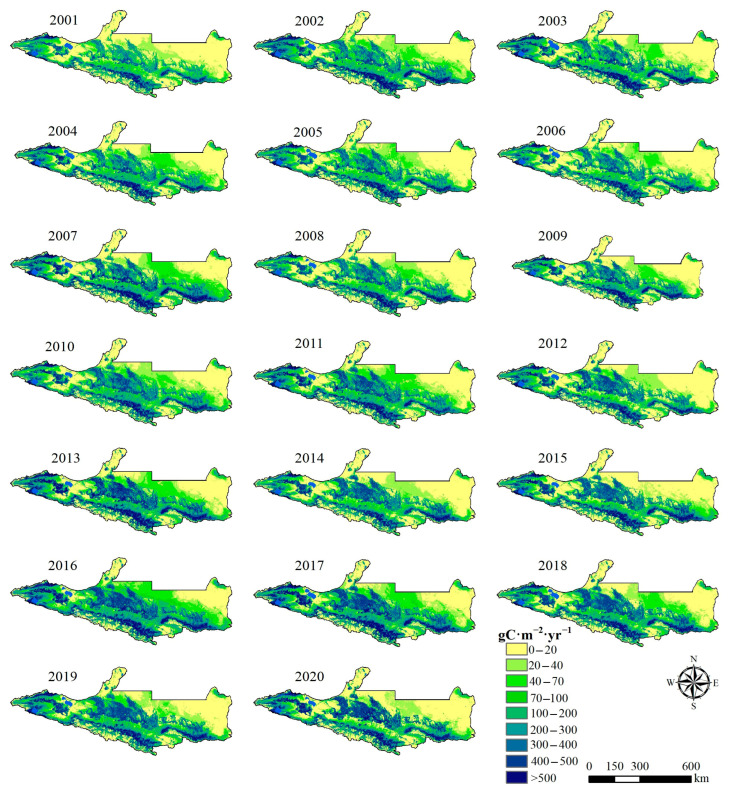
Spatial distribution of NPP on the NSTM from 2001 to 2020.

Figure 2 shows the spatial distribution of vegetation NPP on the NSTM from 2001 to 2020, it can be seen from this that the spatial distribution of NPP in the study area has varied significantly over the past 20 years. The spatial distribution pattern of the NPP was generally characterized as high in the south and low in the north, and high in the west and low in the east. In the western part of the study area, the high-value areas were mainly distributed in the central and western areas of Wenquan County and Bole City, and the low-value areas were mainly distributed in the northern part of Jinghe County and Alashankou City. The high-value NPP areas in the central and eastern parts of the study area were mainly distributed in cultivated land and along the Tianshan Mountains, and the low-value areas were mainly distributed in the northern desert area. Therefore, the spatial distribution of NPP in the study area was closely related to vegetation type, climate characteristics, and topography. In general, mountainous areas and oasis agricultural areas were the main supply areas for NPP on the NSTM, and also the main distribution areas for typical pastures and crop planting on the NSTM, where the unique natural environment provides relatively suitable growth conditions for vegetation, thereby allowing more vegetation to be converted into organic matter through photosynthesis.

**Figure 3:**
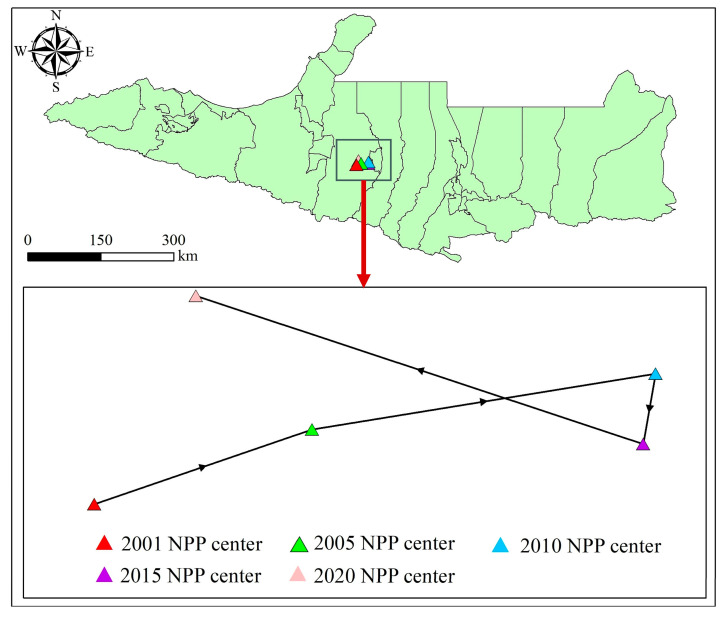
Migration trajectory of the center of gravity for NPP on the NSTM from 2001 to 2020.

The center of gravity of NPP refers to the spatial distribution center of NPP in the study area, which can reflect the spatial concentration trend of NPP, and its position change can reflect the temporal and spatial variation law of NPP [30]. Figure 3 shows the trajectory of the center of gravity for NPP on the NSTM from 2001 to 2020. During the study period, the center of gravity for NPP was always located in Shawan City in the middle of the NSTM, but its location changed during the study period of 20 years. From 2001 to 2005, the center of gravity for NPP moved northeastward by a distance of 10,187.26 m. From 2005 to 2010, the center of gravity continued to move northeastward by 15,399.43 m and reached its easternmost position in 2010, which may have been due to agricultural activities including irrigation and mechanization leading to a significant increase in NPP in the central part of the study area [10]. From 2010 to 2015, the center of gravity for NPP shifted southwestward but it remained relatively stable. From 2015 to 2020, the center of gravity shifted southeastward by 20,838.52 m, which may have been related to the ecological improvement measures implemented in the region.

**Figure 4:**
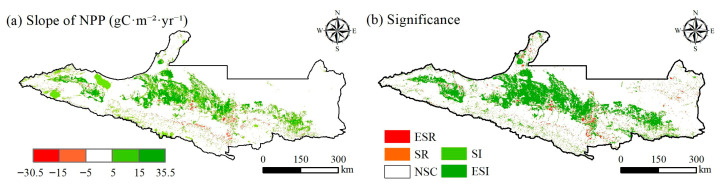
Changing trends in NPP on the NSTM from 2001 to 2020 (**a**) and significance test results (**b**). (ESR, extremely significant reduction; SR, significant reduction; NSC, no significant change; SI, significant increase; ESI, extremely significant increase).

Trend analysis uses linear regression to calculate the slope of the NPP of each grid, the Mann–Kendall test is a commonly used non-parametric statistical analysis tool that is not affected by missing values and outliers and is often used to test the significance of changing trends. The changes in NPP on the NSTM from 2001 to 2020 were studied by conducting trend analysis and Mann–Kendall tests (Figure 4). During the study period, the NPP in the area exhibited an overall upward trend, increasing in 67.50% of the area, and the trend was only downward in 32.50% of the area. In particular, the parts with extremely significant increases and significant increases accounted for 20.96% of the study area, where they were mainly distributed in the oasis plain area, with a small amount distributed in the southern area of the mountain grassland. The significant increase in NPP in the oasis plain area may have been due to agricultural activities, including irrigation and fertilization, and the increase in mountain grassland may have been a consequence of snow melting and increased precipitation caused by global warming. The parts with extremely significant decreases and significant decreases accounted for 1.87% of the study area, where they were mainly distributed in the construction land expansion area, probably because of the surface changes caused by urbanization leading to a decrease in NPP.

### 2.2. Impacts of Land Use Change and Climate Change on NPP

#### 2.2.1. Impacts of Land Use Change on NPP

**Figure 5:**
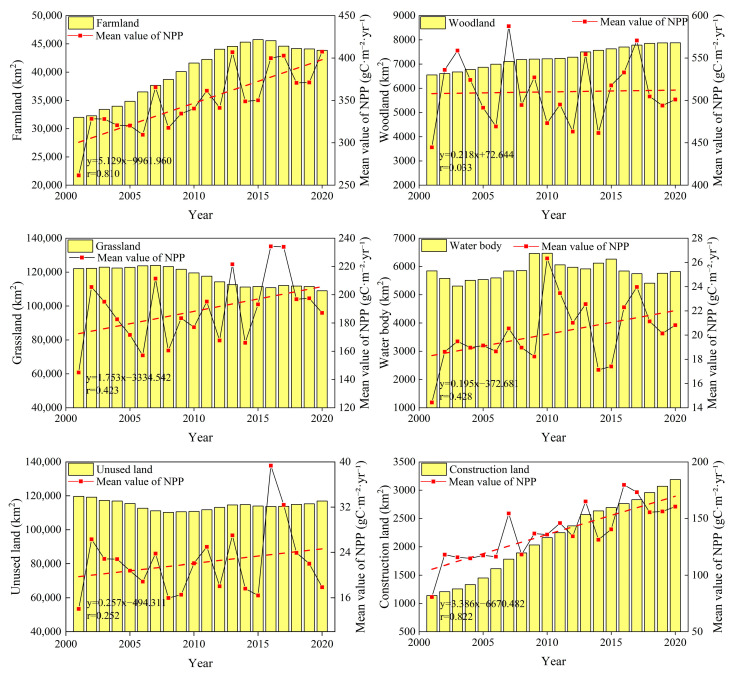
Interannual trends in land use types and annual average NPP on the NSTM from 2001 to 2020.

The land use changes on the NSTM were analyzed from 2001 to 2020. As shown in Figure 5, the farmland area exhibited linear growth from 2001 to 2015, with rapid expansion from 32,009.00 km^2^ to 45,747.50 km^2^, and the area increased by 37.05% year-on-year. However, after 2015, due to the introduction of the ecological protection red line, there was a greater focus on ecological protection and restoration in the region, and the implementation of the “land withdrawal and water reduction” policy led to a downward trend in the farmland area [56]. The area of woodland tended to increase continuously over 20 years, rising from 6551.75 km^2^ in 2001 to 7877.25 km^2^ in 2020, with a year-on-year increase of 20.23%. The area of grassland generally tended to decline from 2001 to 2020, where the area decreased from 122,076.75 km^2^ to 109,011.00 km^2^, with a total reduction of 13,065.75 km^2^ in 20 years. As a consequence of rapid urbanization and economic development, the area of construction land continued to grow from 2001 to 2020, with a growth rate of 179.88%. The area of unused land tended to decrease from 2001 to 2020, with a total reduction of 2757.75 km^2^ in 20 years, thereby indicating that unused land was continuously developed and utilized over the years. During the study period, the area of water body fluctuated significantly, reaching a maximum of 6466.50 km^2^ in 2015, before tending to decrease subsequently.

In general, the average NPP values were relatively high for woodland and farmland, and thus their rapid growth in the area significantly increased the NPP. In particular, the large-scale reclamation of farmland in oasis plains was the main reason for the increased NPP in the region. Large-scale grassland degradation led to the decline in NPP to some extent, but the average NPP of grassland was lower than that of woodland and farmland, so the reduction in the amount of NPP due to grassland was much smaller than the increase in NPP caused by the expansion of woodland and farmland. Due to the small area of construction land, urban expansion had little impact on the overall changes in NPP. The area of unused land fluctuated greatly but its impact on NPP was minimal due to its low carbon fixation capacity. The area of water body fluctuated but the overall change was small during the study period, and thus the contribution to the changes in NPP was also limited.

#### 2.2.2. Changes in NPP Due to Land Use Changes

**Figure 6:**
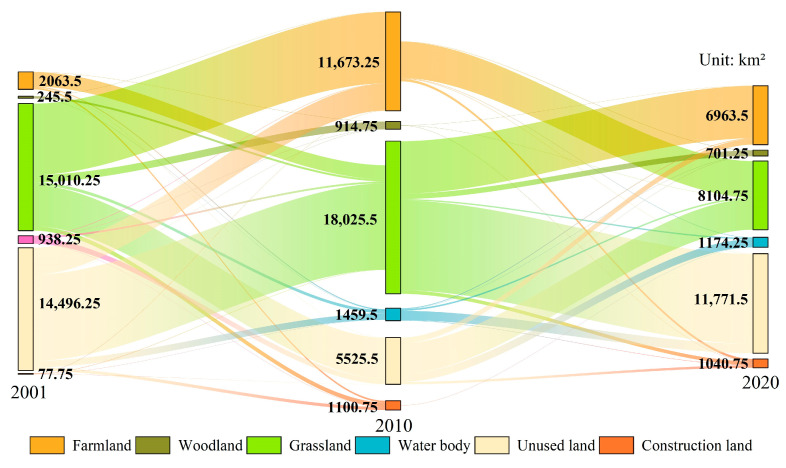
Sankey diagram of land use changes on the NSTM from 2001 to 2020.

Based on the relationships between different land use types on the NSTM from 2001 to 2020 (Figure 6) and the average NPP value for each type of land (Figure 5), we estimated the changes in the total NPP under land use changes in the study area. The results showed that the changes in the areas of land use types on the NSTM from 2001 to 2010 and from 2010 to 2020 accounted for 11.30% and 10.25% of the total area, respectively, with overall decreases in the areas of grassland, unused land, and water body, and significant increases in the areas of farmland, woodland, and construction land. From 2001 to 2010, the area of grassland on the NSTM converted into other land use types was 15,010.25 km^2^, and the area of other land use types converted into grassland was 12,360.25 km^2^. The net increase in grassland NPP of 0.01 TgC was mainly due to the conversion of 10,188.00 km^2^ of unused land to grassland. The average NPP values for unused land and grassland were quite different, where the increase in NPP after the conversion of unused land into grassland compensated for the loss of NPP caused by the large-scale conversion of grassland into farmland. From 2010 to 2020, the area of grassland converted into other land use types was 18,025.50 km^2^, and the area of other land use types converted into grassland was 8104.75 km^2^, resulting in a net decrease of 1.67 TgC in the grassland NPP. The areas of land converted into farmland from 2001 to 2010 and from 2010 to 2020 were 41,633.50 km^2^ and 6963.50 km^2^, respectively, and the areas were much larger than the areas lost, resulting in net NPP increases of 3.43 TgC and 1.23 TgC. The unused land transfer-out areas from 2001 to 2010 and from 2010 to 2020 were 14,496.25 km^2^ and 5524.50 km^2^, respectively, and the transfer-in areas were 4884.25 km^2^ and 11,771.50 km^2^, resulting in a net decrease of 0.08 TgC in the NPP of unused land from 2001 to 2010, and a net increase of 0.09 TgC in the NPP from 2010 to 2020. In both study periods, the area changed into woodland was larger than the area lost, resulting in net increases in NPP of 0.32 TgC and 0.34 TgC, respectively. Construction land maintained steady growth over the years, with less conversion into other land types, and it was mainly changed from other land types, where the areas gained in the two study periods were 1100.75 km^2^ and 1040.75 km^2^, respectively, resulting in net increases of 0.14 TgC and 0.17 TgC in the NPP of construction land. From 2001 to 2010, the area of water bodies gained was larger than the area lost, resulting in a slight increase of 0.02 TgC in the NPP of water body. From 2010 to 2020, the area of water body gained was smaller than the area lost, resulting in a net NPP decrease of 0.01 TgC.

#### 2.2.3. Impacts of Climate Change on NPP

**Figure 7:**
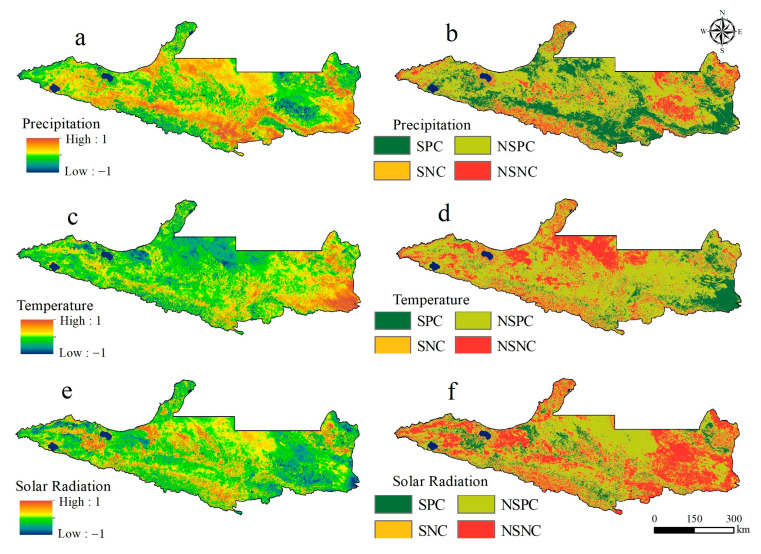
Spatial distribution of partial correlation coefficients (**a**,**c**,**e**) and correlations that passed the significance test (**b**,**d**,**f**) between different climate factors and NPP (SPC, significant positive correlation; NSPC, not significantly positively correlated; SNC, significant negative correlation; NSNC, not significantly negatively correlated).

Climate change directly affects photosynthesis, respiration, transpiration, and other metabolic processes in vegetation, which then affect the changes in vegetation NPP [35,57,58]. As shown in Figure 7, significant spatial heterogeneity was found between vegetation NPP and climate factors (precipitation, temperature, and solar radiation), and vegetation NPP was generally positively correlated with meteorological factors. The partial correlation coefficient between vegetation NPP and precipitation was 0.30, and the areas with positive correlations accounted for 85.80% of the areas, where 29.18% were extremely significantly positively correlated, where they were mainly distributed in mountainous areas with higher altitudes in the south and desert areas in the north. The impacts of human activities were relatively low in these areas, so the increase in precipitation had a significant impact on the growth of vegetation. In addition, 14.20% of the areas were negatively correlated, and the areas with significant negative correlation accounted for only 0.25%, where they were scattered in unused land areas in the east and south of the study area. The partial correlation coefficient between vegetation NPP and temperature was 0.17, and the positive correlation accounted for 75.75% of the study area, where the areas with significant positive correlations accounted for 11.24%, which were mainly concentrated in woodland and grassland areas in the east and south of the study area and farmland areas in the west. The areas with negative correlations accounted for 24.25%, where the areas with significant negative correlation accounted for only 0.18%, which were mainly distributed in the snow-covered areas at high altitudes in the Tianshan Mountains and the edge of the desert area. The partial correlation coefficient between vegetation NPP and temperature was 0.09, and 62.96% of the areas were positively correlated, where 8.78% were significantly positively correlated and mainly concentrated in farmland areas of the oasis and woodland and grassland areas at higher altitudes. In addition, 37.04% of the areas were negatively correlated, where 1.37% were significantly negatively correlated and scattered in the west and east of the study area. In summary, the correlation was highest between the NPP of vegetation on the NSTM and precipitation, and increases in precipitation, temperature, and solar radiation promoted increases in the NPP of mountainous and plain areas.

### 2.3. Analysis of NPP Driving Factors

#### 2.3.1. Identification of OPGD Model Parameters

**Figure 8:**
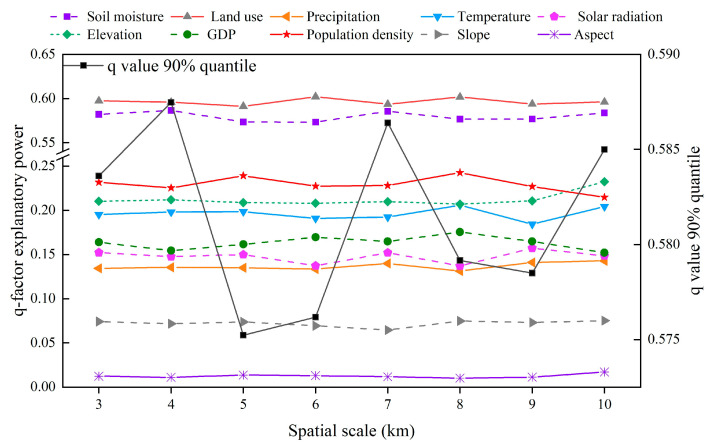
Explanatory power of factors at different spatial scales and their 90% quantiles.

**Figure 9:**
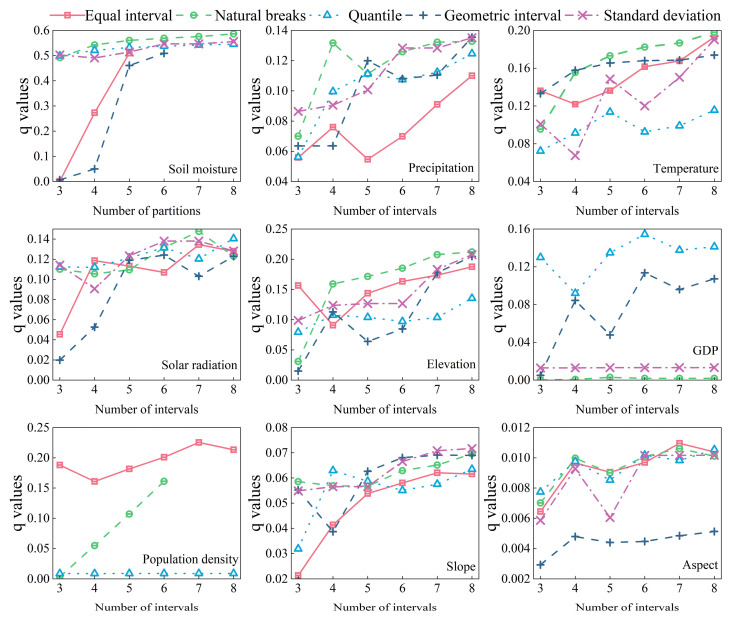
Optimal discretization method and partition number identification of some influencing factors.

As shown in Figure 8, among the eight spatial scales, the 90% quantile of the explanatory power of each factor tended to fluctuate in a downward manner. The q value peaked at 4 km, so a 4 km mesh was constructed as the sampling grid for each factor. The differences in the explanatory power of each factor under different combinations of discretization methods and numbers of partitions were then identified (Figure 9). Considering precipitation as an example, the explanatory power was highest for this factor when the data discretization method was the geometric interval method, and the number of partitions was eight. Therefore, the geometric interval method was used to divide precipitation into eight categories. Similarly, based on the maximum q value principle, the optimal discretization method and number of partitions were then determined for each factor (Figure 9).

#### 2.3.2. Single Factor Analysis of Influencing Factors

**Figure 10:**
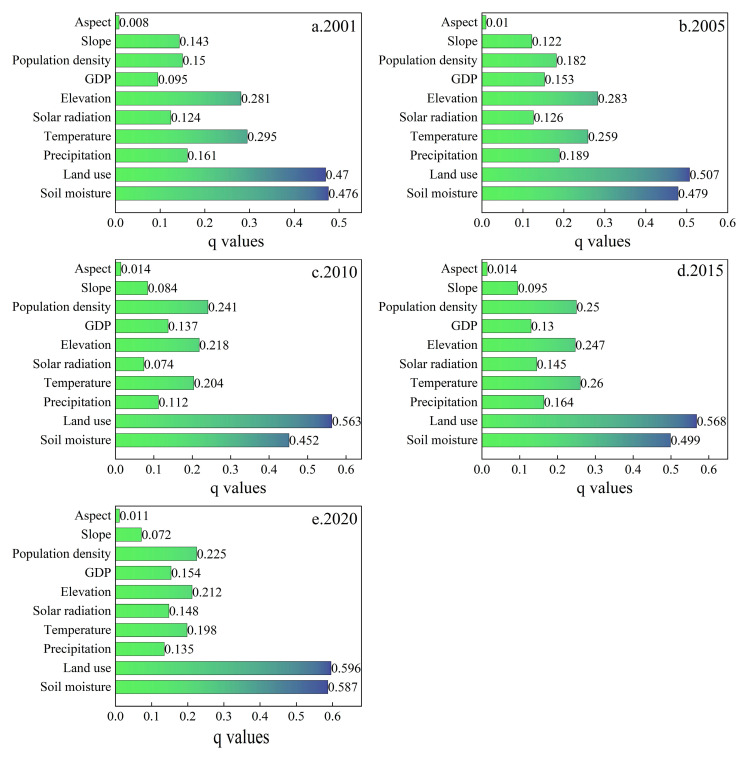
Detection of single factors that affected NPP of vegetation on the NSTM from 2001 to 2020.

Based on the obtained optimal parameters of the OPGD, single factor detection was used to clarify the explanatory power of each influencing factor on NPP in the five periods of 2001, 2005, 2010, 2015, and 2020 (Figure 10). The results show that from 2001 to 2020, land use type and soil moisture have a strong explanatory power for the NPP in the study area, this shows that land use type and soil moisture are the main factors affecting NPP. In addition, temperature, elevation, and population density all show relatively significant explanatory power for NPP; the explanatory power of slope and aspect on NPP is always less than 0.13, indicating that their impact on NPP is small. Over 20 years, the explanatory power of each factor fluctuated to varying degrees, where the explanatory powers for land use type (0.596) and soil moisture (0.587) were 0.13 and 0.11 higher, respectively, in 2020 compared with 2001, indicating the increasing impacts of land use type and soil moisture on NPP. In addition, the explanatory powers tended to increase for GDP and population density, and thus the impacts of human activities on NPP gradually increased over the years. The explanatory powers fluctuated to varying degrees for precipitation and temperature, indicating significant correlations between dramatic changes in climate conditions and NPP. In summary, the land use type and population density were factors with high explanatory power among human activities, while the temperature had high explanatory power among climate conditions, and elevation and soil moisture had high explanatory power among natural environment factors. Thus, the spatial differentiation of vegetation NPP in the region was jointly dominated by human activities, climate change, and natural environment factors.

#### 2.3.3. Interaction Detection of Influencing Factors

**Figure 11:**
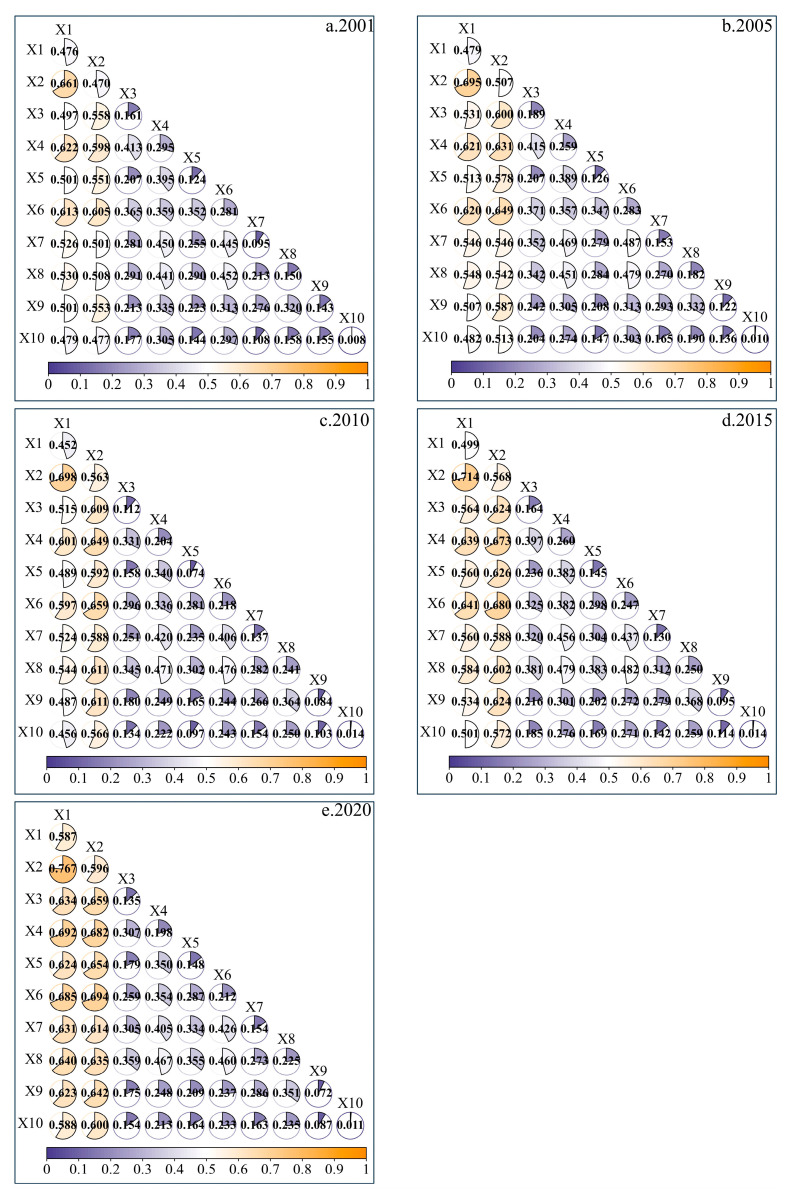
Interactions between factors that affected the vegetation NPP on the NSTM from 2001 to 2020 (X1, soil moisture; X2, land use; X3, precipitation; X4, temperature; X5, solar radiation; X6, elevation; X7, GDP; X8, population density; X9, slope; X10, aspect).

In order to reveal the effect of interaction between factors on NPP, this study uses interaction detection to obtain the explanatory power of interaction between driving factors in 2001, 2005, 2010, 2015, and 2020 (Figure 11). The detection results show that the explanatory power of interaction between factors on NPP is generally greater than the effect of a single factor, and the interaction results mostly show different degrees of double factor enhancement and nonlinear enhancement.

During the entire period, the most powerful interaction between factors that explained changes in the vegetation NPP on the NSTM in 2001, 2005, 2010, 2015, and 2020 was soil moisture ∩ land use type; thus, human interference acting on natural environmental conditions had a significant effect on vegetation NPP in the study area. In addition, the explanatory power of the interaction between temperature and other factors in the five periods ranked in the top three, indicating that the interaction between temperature and other factors in different periods was most important for influencing the vegetation NPP in the study area. In the five periods, the explanatory power of the interaction between elevation and other factors ranked in the top four, indicating that the interaction between elevation and other factors in each period had significant effects on the vegetation NPP. Analysis of the effects of interactions between factors in different years on the explanatory power for changes in NPP showed that the mean explanatory powers of slope and aspect from 2001 to 2020 were only 0.103 and 0.011, respectively, but after interacting with other factors, their mean explanatory powers increased significantly to 0.341 and 0.264. In general, single factors with weak explanatory power for changes in the NPP could have significant effects on the vegetation NPP after interacting with other factors with high explanatory power.

### 2.4. CASA Model Validation

**Figure 12:**
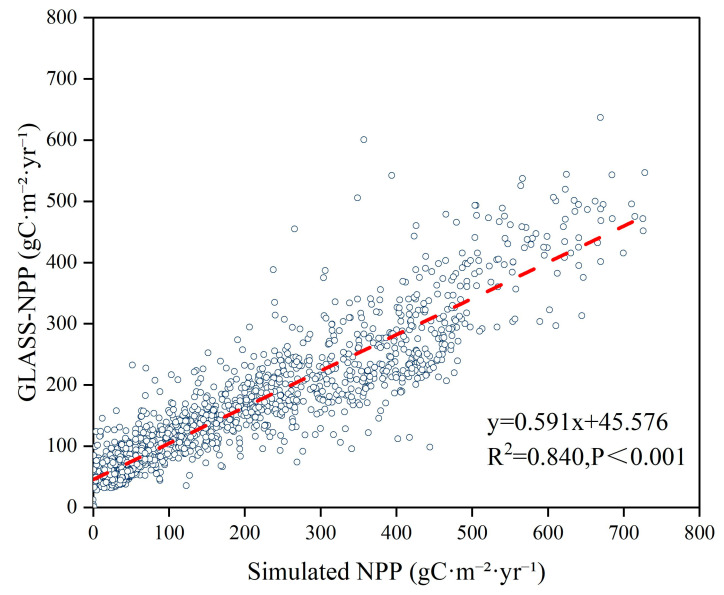
Comparison and verification of simulated NPP values and GLASS-NPP data.

In order to evaluate the accuracy of the CASA model, GLASS–NPP annual remote sensing data set and data from previous studies were used to evaluate the NPP estimates. As shown in Figure 12, the simulated data were strongly correlated with the remote sensing product data, where the relationship was y = 0.591x + 45.567 (R^2^ = 0.840, *p* < 0.001). However, the average NPP estimated by the CASA model (149.43 g·C^−1^·m^−2^) differed from the GLASS product data (156.12 g·C^−1^·m^−2^); the difference in average values is 6.69 g·C^−1^·m^−2^, and this difference is mainly due to the difference in model calculation methods. To avoid differences in modeling methods, we compared the results obtained in previous studies using the CASA model and our results, which showed that our estimated NPP values were similar to those reported in previous studies (Table 1). However, there were still some differences, mainly because of differences in the model parameters, meteorological data acquisition methods, and vegetation type classifications, and the type of NDVI data set also affected the NPP estimated [59]. Thus, the CASA model estimation results were highly reliable and suitable for application in studies of the vegetation NPP on the NSTM.

## 3. Discussion

### 3.1. Advantages of the OPGD Model

The use of different methods for quantifying the impacts of various factors on NPP will lead to certain deviations in the quantification results, e.g., using residual analysis methods to quantify the impacts of climate change and human activities on NPP in some regions may overestimate the contributions of human activities [64]. Due to the limited scope of human activities, it is impossible for them to affect the vegetation in each pixel. Therefore, a regional separation method was proposed to divide vegetation into areas affected by climate change and areas affected by both climate change and human activities [10,65]. In addition, linear relationship methods, such as correlation and regression analysis methods, were used in some studies to quantify the dynamic relationships between environmental variables and vegetation [35,49], but all of these methods assume that there are significant linear relationships between specific factors and changes in vegetation. However, the relationship between the vegetation NPP and climate is not simply linear, so we used the OPGD model to quantify the effects of driving mechanisms to climate change and human activities, and changes in the vegetation NPP on the NSTM. This model is an improved version of the traditional GDM that avoids subjective effects due to the spatial scale and data discretization processing to identify the optimal combination of the spatial scale, spatial data discretization method, and number of partitions in order to explore the effects of single factors and combinations of two factors on NPP from the perspective of spatiotemporal heterogeneity.

### 3.2. Response of Vegetation NPP to Human Activities

Human activities affect the NPP of terrestrial ecosystems in various ways (including positive and negative effects), but land use change is considered the most important factor related to human activities [20,66]; therefore, this paper discusses the changes in NPP caused by direct human activities. Our results are consistent with those obtained in previous studies in this area [10,60], which also showed that land use change significantly affected the vegetation NPP. Land use change is an important factor that affects changes in vegetation cover, and the form and intensity of land use by human activities have continued to increase with the implementation of urbanization and ecological projects, thereby leading directly to changes in surface vegetation cover to then affect the regional vegetation NPP [35,67,68]. Many studies have shown that urbanization is negatively correlated with vegetation NPP [69,70]. However, we found that even in the context of rapid urbanization, the NPP on the NSTM exhibited a fluctuating upward trend (Figure 1), which can be explained by the following two main reasons. First, the unique natural geographical conditions on the NSTM have a positive effect on the stability of natural vegetation, where the geographical features of the region consist of mountainous areas, oasis areas, and desert areas from south to north. Land use changes in the mountainous areas were mainly affected by climate change, where the area of ice and snow gradually shrank due to the increase in temperature over the years, but the overall land use structure remained basically stable in the mountainous areas (Figure S1) [56], thereby positively affecting the vegetation NPP. Second, oasis areas were the main locations for agricultural cultivation and economic development, and land use changes were significantly affected by human activities in these areas. As shown in Figure S1, from 2001 to 2020, Xinjiang has begun to develop water-saving irrigation technology and improve agricultural mechanization on a large scale [71]. Urbanization and industrialization developed rapidly with the implementation of the “Ninth Five-Year Plan” and “Western Development Policy” in the region, where the social and economic levels increased rapidly, and the influx of population increased significantly, and thus the land area urgently required for food production and living purposes had to be increased. The oasis plain area was reclaimed and cultivated on a large scale, and the areas of grassland and unused land decreased significantly, with increases in the areas of cultivated land and construction land of 11,858.50 km^2^ and 2057.00 km^2^, respectively (Figure S1). The natural oasis areas were gradually transformed into artificial oasis areas, where the availability of water, warm environment, and appropriate fertilization measures promoted the growth of vegetation [72,73]. In addition, the increased use of water for irrigation changed the effectiveness of the soil moisture and promoted photosynthesis, nutrient transport, and other processes [74], with a positive effect on the NPP of oasis vegetation, it can be seen that the increase in NPP on the NSTM was mainly due to the large-scale conversion of other land use types into farmland, where this process was mainly driven by human activities such as farmland expansion, and it was the main contributor to the increase in NPP in the study area, as also shown in previous studies [10]. The desert areas outside the oasis are threatened by salinization and desertification, with poor ecosystem stability and the vegetation NPP is low, with great variation.

### 3.3. Response of Vegetation NPP to Climate Change

**Figure 13:**
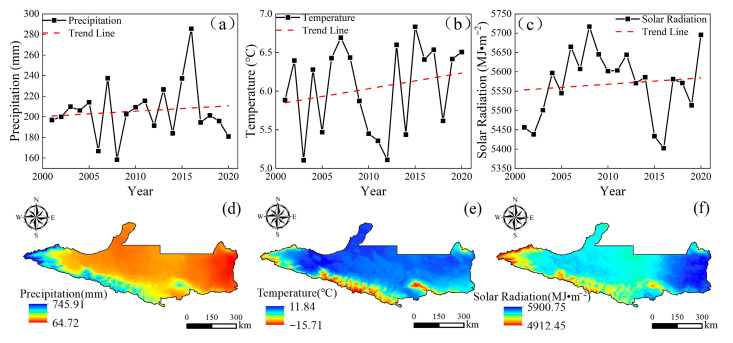
Interannual variations (**a**–**c**) and spatial distributions (**d**–**f**) of climate factors (precipitation, temperature, and solar radiation) on the NSTM from 2001 to 2020.

Many studies have shown that the three main climatic factors that determine the NPP of vegetation in arid areas are precipitation, temperature, and solar radiation [35,60,75], where the influence of precipitation is particularly significant. Precipitation can replenish the soil moisture, alleviate water stress, and increase stomatal conductance to enhance the absorption of carbon dioxide by vegetation [45,76]. This study is consistent with the results of previous studies. Figure 13 shows the mean changes and spatial distribution patterns of the three climate factors on the NSTM from 2001 to 2020. As shown in Figure 13d, the average annual precipitation in the study area gradually decreased from south to north, which was highly consistent with the spatial distribution of the vegetation NPP in areas without human activity. The NSTM is located in the arid northwest region of China, where the water supply limits vegetation growth. Throughout the entire study period, the annual precipitation exhibited a fluctuating upward trend, indicating that the region was generally becoming “wetter” (Figure 13a), thereby enhancing physiological metabolic processes in vegetation, and promoting plant growth and development to increase the vegetation NPP. As shown in Figure 13e, the temperature was characterized as high in the north and low in the south, which was the opposite of the characteristic spatial distribution of vegetation. Over the past 20 years, the temperature in the study area has generally followed a fluctuating upward trend, indicating that the region is “warming” (Figure 13b). Figure 7 also shows that the increased temperature has contributed to the increased NPP in the study area, although the effect of temperature on the vegetation NPP was relatively weak compared with that of precipitation. Alpine ice and snow and seasonal snowmelt are important sources of water in Xinjiang, and rising temperatures have led to glaciers and snow melting, forming larger and earlier river runoffs [77,78]. These abundant water resources have significantly enhanced the vegetation NPP in the mountainous and plain areas, whereas rising temperatures have increased the soil moisture evaporation rate in the northern desert area to largely offset the positive effects of rising temperatures on plant photosynthesis [79], thereby negatively affecting the vegetation NPP. As shown in Figure 13f, the solar radiation on the NSTM was low in the south and high in the north, high in the east, and low in the west, and the solar radiation generally tended to increase during the study period (Figure 13c). Figure 7 also shows that an appropriate increase in solar radiation could promote photosynthesis by vegetation to affect the accumulation of plant organic matter and accelerate the development of plants, and thus the solar radiation was basically positively correlated with the vegetation NPP in the central and western parts of the study area. However, excessively high intensity solar radiation will lead to the photoinhibition of vegetation, thereby increasing the intensity of transpiration and accelerating the loss of water, and ultimately lead to a decrease or even the cessation of productivity by vegetation, with negative impacts on normal plant growth and development [61]. Therefore, the vegetation NPP in most areas of Jimusar, Qitai County, and Jimusar County was negatively correlated with solar radiation.

### 3.4. Effects of Topographic Factors on Vegetation NPP

Topographic factors affect the spatial distribution of vegetation by affecting water, heat, and nutrients [80]. The NSTM has a typical “mountain–basin” system structure with complex terrain consisting of mountains of various sizes, and the vegetation types generally follow a vertical distribution from north to south as the altitude increases. This unique geographical environment determined the spatial distribution and characteristics changes in the vegetation NPP in the study area. As shown in Figure S2, the NPP tended to increase initially and then decreased as the altitude increased. The area below 1000 m above sea level mainly consisted of natural grassland and crops, and the NPP ranged between 17.96 and 201.07 gC⋅m^−2^⋅yr^−1^. The solar radiation and precipitation increased as the altitude increased in the range of 1000–2400 m, and the negative impacts of human activities on vegetation were lower, and thus the growth condition of vegetation and mean NPP increased gradually. The NPP increased the most as the altitude increased in the range between 1650 and 2150 m. The climate conditions were harsh at altitudes exceeding 2400 m and the thick snow in the Tianshan Mountains made the area unsuitable for the growth of natural vegetation, and thus the NPP decreased [65]. As the slope increased, the NPP value generally increased initially and then decreased, where the average NPP in the area with a slope less than 3° was 110.81 gC⋅m^−2^⋅yr^−1^, which mainly consisted of farmland, grassland, and unused land. The area with slopes ranging between 3 and 14° mainly contained grassland and woodland, and the vegetation NPP increased significantly as the slope increased. The maximum NPP value was found when the slope was 14°, but soil erosion increased when the slope exceeded 14° and the capacity to retain water and fertilizer decreased, and thus the vegetation NPP also tended to decrease [81].

### 3.5. Limitations and Prospects

The CASA model has been widely used in previous studies of NPP estimation for vegetation in arid areas, and thus the model is highly suitable for application in arid areas [82,83], although there are some uncertainties in the model construction process. In the present study, due to the lack of data measurements for some model parameters, we used the maximum LUE and other parameters for different vegetation types reported by Zhu et al. [33] to estimate NPP. These parameters were obtained based on the actual situation for vegetation types in China, where they have been shown to be suitable, but there may have been some small uncertainties. In addition, the meteorological data (such as temperature, precipitation, and solar radiation) used in this study were based on remotely sensed meteorological raster data, which may have led to potential uncertainties [84], but such uncertainties are inevitable in large-scale studies [20]. We analyzed the spatiotemporal changes and driving mechanism to the vegetation NPP on the NSTM at a high spatial resolution and long time scale, thereby providing a theoretical basis for regulating vegetation carbon sequestration in future studies. The NPP estimation process and input meteorological data may produce certain uncertainties but, in this study, we used an effective and feasible method for quantifying the impacts of climate and human activities on the changes in NPP on a large scale and analyzing the nonlinear relationships between NPP and driving factors from the perspective of spatiotemporal heterogeneity, which are not possible using principal component analysis, traditional GDM, and structural equation models. In future studies, more representative sample plots can be established to obtain model parameters for specific localities. In addition, ground observation data, optimal spatial interpolation methods, and reanalysis data can be effectively combined to reduce the uncertainty regarding meteorological input data.

In this study, we analyzed the driving mechanism to changes in the vegetation NPP according to quantitative and spatial heterogeneity perspectives, but our method also had some limitations because we identify climate change, human activities, and geographic environmental characteristics as the main drivers of NPP changes. However, in reality, factors such as forest fires [85], overgrazing [68], biological disasters [86], and extreme climate [87] can all affect the vegetation NPP, but they were not quantified in this study. These events may be indirectly attributed to climate change and human activities, but distinguishing their specific effects on NPP remains a challenge for future research. In addition, increases in the atmospheric CO_2_ concentration and nitrogen deposition can promote the fertilization of vegetation, and they have crucial effects on the changes in NPP [88,89]. Therefore, in future research, it is important to explore the impacts of increases in the CO_2_ concentration and nitrogen deposition in arid areas on changes in the vegetation NPP.

## 4. Materials and Methods

### 4.1. Overview of the Study Area

**Figure 14:**
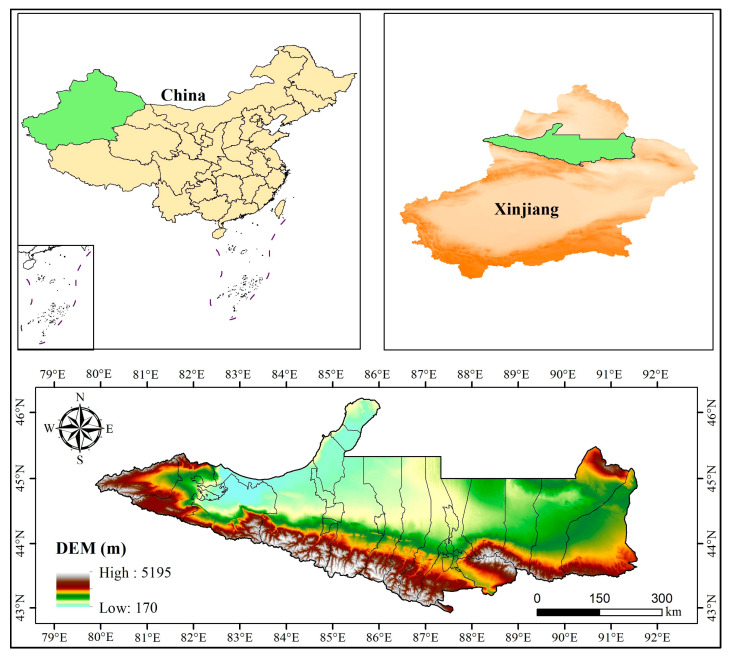
Location and overview of the study area.

The NSTM are located in Xinjiang, China, in the heart of the Eurasian continent, on the southern edge of the Junggar Basin (Figure 14). The NSTM mainly consist of Urumqi City, Shihezi City, Karamay City, Changji City, Fukang City, Hutubi County, Manas County, Kuitun City, Shawan City, Wusu City, and Bole City, etc., which contain 77.60% of the heavy industry and 66.50% of the light industry in the whole territory, with the highest concentrations in Xinjiang in terms of population, industry, and economy. The region has a temperate continental climate, with an arid climate, four distinct seasons, abundant light but large temperature differences, and low precipitation and high evaporation, where the average annual precipitation is 20–400 mm and the evaporation is up to 1817 mm. The study area extends from the Tianshan Mountains in the south to Gurbantunggut Desert in the north, with an oasis plain in the center. The terrain gradually decreases from south to north, forming a unique “mountain–oasis–desert” complex ecosystem, where the mountainous area is the flow-producing area of the region, the oasis area is the water resource consumption area, and the desert area is the water resource dissipation area. Due to its unique geographic location, this region has become a strategic area connecting China with Central Asia and Europe, a land transportation hub for cooperation and exchange in China’s Central and West Asia regions, and a core area of the Silk Road Economic Belt [90].

### 4.2. Data Sources and Processing

The remote sensing data used for this study included NDVI, vegetation type data, and NPP products. As shown in Table 2, the NDVI data were derived from the MCD13Q1 data set at a spatial resolution of 500 m. Updating every 16 days, cloud removal, projection transformation, and normalization were performed using Google Earth Engine to obtain the monthly NDVI data set based on the maximum value synthesis method. The MCD12Q1 data use the vegetation classification scheme of the International Geosphere–Biosphere Program and contain 17 vegetation types, including forests, grasslands, agricultural lands, and towns. The GLASS–NPP data were obtained from the Global Land Surface Satellite (GLASS) data set of Beijing Normal University, with a spatial resolution of 500 m and temporal resolution of 1 year. These data were obtained based on calculations using the EC–LUE model, and the accuracy of the estimated data was found to be reliable [60]. A year-by-year NPP data set for the study area from 2001 to 2020 was obtained by mosaicking, projection, and resampling.

Meteorological data (temperature, precipitation, and solar radiation) were month-by-month raster data, where monthly averages were used for temperature data and monthly cumulative values for precipitation and radiation data. As shown in Table 2, the raster data were processed by batch projection transformation, resampling, and extraction by masking using the model builder in ArcGIS 10.8 to obtain the month-by-month data set for the study area, which was synthesized and cropped from year-to-year to obtain the annual data series.

The other data sets mainly included land use data, socio-economic data, topographic, and geomorphological data. In particular, the land use data for 2001 to 2020 came from the China Land Cover Data Set provided by Professors Jie Yang and Xin Huang, which classifies land into nine categories with an accuracy of more than 85% [60]. We reclassified the data into six categories: farmland, woodland, grassland, water body, unused land, and construction land according to the China Land Resources Classification System standards and the needs of this study [20,91]. The unit for gross domestic product (GDP) data was million yuan per square kilometer with a spatial resolution of 1000 m, and the unit for population data was people per square kilometer with a spatial resolution of 1000 m [30]. Slope and aspect data for the study area were obtained by processing the DEM data using the Slope and Aspect functions in ArcGIS 10.8.

All data were reprojected using the WGS_1984_World_Mercator coordinate system and resampled to a spatial resolution of 500 m.

### 4.3. Research Methodology

**Figure 15:**
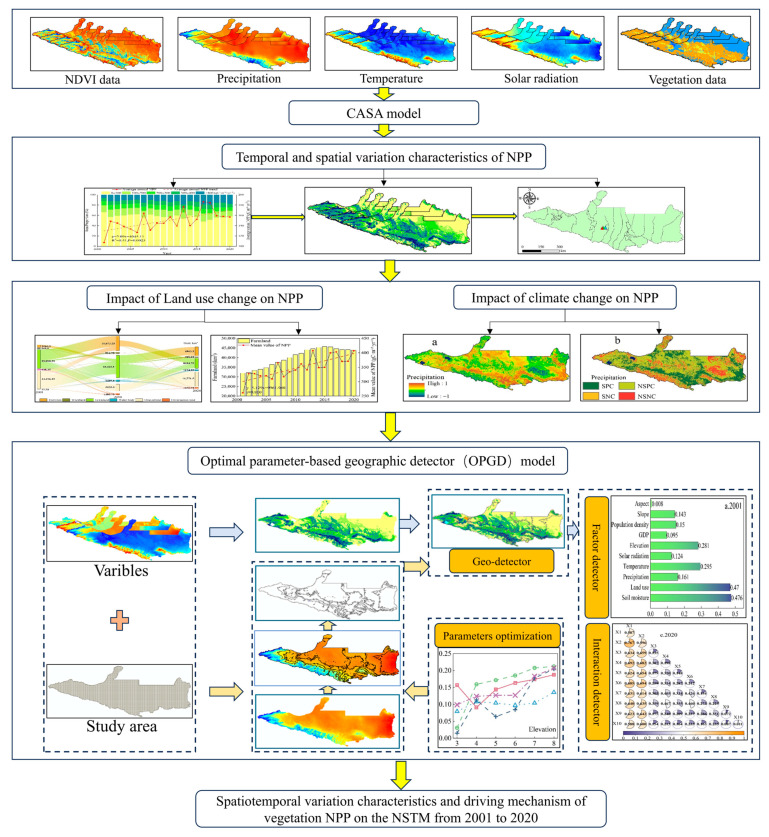
Flow chart illustrating the research process.

The research process is illustrated in Figure 15. First, the CASA model was driven by the meteorological data, vegetation type data, NDVI data, and model parameters to estimate the NPP of vegetation on the NSTM from 2001 to 2020, and the spatiotemporal distribution and changes in NPP were analyzed. Second, based on the spatiotemporal variations in land use, the impacts of human activities on NPP were statistically analyzed, and partial correlation analysis was conducted between the NPP data and meteorological data to determine the impacts of climate change on NPP. Finally, the OPGD model was used to comprehensively elucidate the mechanisms that allowed different factors to affect NPP based on the optimal spatial scale, discretization method, and combination of partition numbers.

#### 4.3.1. Calculation of NPP Using the CASA Model

The CASA model calculates vegetation NPP based on the absorbed photosynthetically active radiation (APAR) and actual light energy use efficiency (LUE). Environmental factors such as soil moisture, solar radiation, and temperature all affect the actual LUE. The CASA model considers the vegetation growth status and environmental stress factors, and it has been widely used for global NPP estimation and is well validated [92]. In this study, we applied the improved CASA model described by Zhu Wenquan et al. [33] to estimate the vegetation NPP on the NSTM. The model comprehensively considers NDVI, temperature, precipitation, solar radiation, and vegetation type data, as well as the differences in maximum LUE among different vegetation types:

(1) $$NPP\left(x,t\right)=APAR\left(x,t\right)\times \varepsilon \left(x,t\right)$$

where *NPP* (*x, t*) is the net primary productivity for image *x* in month *t*, *APAR* (*x*, *t*) is the amount of photosynthetically active radiation absorbed by the vegetation in image *x* during month *t* ($gC\cdot m^{-2}\cdot {month}^{-1}$), and *ε* (*x, t*) is the actual light energy utilized by the vegetation in image *x* ($gC\cdot {MJ}^{-1}$).

(2) $$APAR\left(x,t\right)=FPAR\left(x,t\right)\times SOL\left(x,t\right)\times 0.5$$

where *FPAR* (*x*, *t*) is the proportion of photosynthetically active radiation absorbed by vegetation in image *x*, *SOL* (*x*, *t*) is the total amount of solar radiation received by image *x* in month *t*$\;\left(MJ\cdot m^{-2}\right)$, and 0.5 is the proportion of solar radiation (wavelength 0.4–0.7 μm) that can be absorbed and utilized by vegetation among the total solar radiation. *FPAR* (*x, t*) is calculated as follows:

(3) $$FPAR\left(x,t\right)=\frac{\left(NDVI\left(x,t\right)-{NDVI}_{i,min}\right)\times \left({FPAR}_{max}-{FPAR}_{min}\right)}{{NDVI}_{i,max}-{NDVI}_{i,min}}+{FPAR}_{min}$$

where *NDVI* (*x, t*) is the *NDVI* value for pixel *x* in month *t*; ${NDVI}_{i,min}$ and ${NDVI}_{i,max}$ are the minimum and maximum *NDVI* values for the i-th vegetation type, respectively; and ${FPAR}_{max}$ and ${FPAR}_{min}$ are constants with values of 0.95 and 0.001.

Previous studies have shown that there is a linear relationship between *FPAR* and the vegetation index ratio (*SR*), and the average *FPAR* value calculated based on the *NDVI* and *SR* methods has the smallest error relative to the actual value. The formulae are as follows:

(4) $$FPAR\left(x,t\right)=\frac{\left(SR\left(x,t\right)-SR_{i,min}\right)\times \left({FPAR}_{max}-{FPAR}_{min}\right)}{SR_{i,max}-SR_{i,min}}+{FPAR}_{min}$$

(5) $$\;SR\left(x,t\right)=\left[\frac{1+NDVI\left(x,t\right)}{1-NDVI\left(x,t\right)}\right]$$

(6) $$\;FPAR=\frac{{FPAR}_{NDVI}+{FPAR}_{SR}}{2}$$

The light energy utilization rate (*ε*) refers to the efficiency of vegetation at converting absorbed effective photosynthetic radiation into organic carbon through photosynthesis. It is mainly determined by temperature and moisture, and is calculated as follows:

(7) $$\varepsilon \left(x,t\right)=T_{\varepsilon 1}\left(x,t\right)\times T_{\varepsilon 2}\left(x,t\right)\times W_{\varepsilon }\left(x,t\right)\times \varepsilon_{\max\;}$$

where $T_{\varepsilon 1}\left(x,t\right),T_{\varepsilon 2}\;\left(x,t\right),W_{\varepsilon }(x,t)$ represent the effects of temperature and moisture on the light energy utilization, respectively; and $\varepsilon_{max}$ represents the maximum light energy utilization by vegetation under ideal conditions, and its value is related to the vegetation type. The value of $\varepsilon_{max}$ used in this study is based on the results reported by Zhu et al. [33] who applied NPP data measurements to calibrate the maximum LUE for different vegetation categories in China.

#### 4.3.2. Trend Analysis and Mann–Kendall Trend Test

According to the changes in the vegetation NPP pixel values from 2001 to 2020, a pixel-based univariate linear regression equation was established to analyze the interannual varying trend in NPP. The slope of the equation represents the annual rate of change in NPP over 20 years:

(8) $$\;\mathrm{slope}\;=\frac{n\times \sum_{i=1}^{n}\;i\times {NPP}_{i}-\left(\sum_{i=1}^{n}\;i\right)\left(\sum_{i=1}^{n}\;{NPP}_{i}\right)}{n\times \sum_{i=1}^{n}\;i^{2}-{\left(\sum_{i=1}^{n}\;i\right)}^{2}}$$

where slope indicates the annual rate of change in *NPP*, *i* is the year, *NPP_i_* is the *NPP* value in year *i*, *n* is the number of years in the study period, and *n* = 20 in this study. *NPP* has an increasing trend when slope > 0 and a decreasing trend when slope < 0.

The Mann–Kendall trend test is a nonparametric statistical test proposed by Mann and Kendall for detecting the changing trend in time series and is typically used to test the significance of the trend, so it was appropriate for determining the trend in the vegetation npp in the present study. The Mann–Kendall statistical trend test method is as follows:

(9) $$S=\sum_{i=n}^{n-1}\;\sum_{j=i+1}^{n}\;sign\left({NPP}_{j}-{NPP}_{i}\right)$$

(10) $$sign\left({NPP}_{j}-{NPP}_{i}\right)=\left\{\begin{array}{c} 1,\;{NPP}_{j}-{NPP}_{i}>0\\ 0,\;{NPP}_{j}-{NPP}_{i}=0\\ -1,\;{NPP}_{j}-{NPP}_{i}<0 \end{array}\right.$$

(11) $$Z=\left\{\begin{array}{c} \frac{s-1}{\sqrt{Var\left(s\right)}},\;\left(s>0\right)\\ 0,\;\left(s=0\right)\\ \frac{s+1}{\sqrt{Var\left(s\right)}},\;\left(s<0\right) \end{array}\right.$$

where *NPP_i_* and *NPP_j_* are the NPP data for year *i* and year *j*, respectively, and *i* < *j*; *n* is the length of the data set, with *n* = 20 in this study; *Z* is a normally distributed statistic, and *Var* (s) is the variance. Significance tests were performed at significance level α. If |Z| ≥ Z_α/2_, the original hypothesis was rejected. When |Z| was greater than 1.96 and 2.58, the trend passed the significance test at the 95% and 99% confidence levels, respectively. In this study, we classified the significance of the trends in the changes in NPP on the NSTM from 2001 to 2020 into five categories according to the specific criteria shown in Table 3.

#### 4.3.3. Center of Gravity Migration Trajectory Model

In this study, the center of gravity migration trajectory model was applied to calculate the location of the center of gravity of the NPP in the study area in 2001, 2005, 2010, 2015, and 2020, and to plot the center of gravity migration trajectory representing the degree of spatial change in NPP and its trend, according to the following calculation [93]:

(12) $$X_{i}=\frac{\sum_{i=1}^{n}\;\left(A_{ji}\times X_{ji}\right)}{\sum_{i=1}^{n}\;A_{ji}}$$

(13) $$Y_{i}=\frac{\sum_{i=1}^{n}\;\left(A_{ji}\times Y_{ji}\right)}{\sum_{i=1}^{n}\;A_{ji}}$$

where *X_i_*, *Y_i_* are the latitude and longitude coordinates of the center of gravity of the NPP distribution in year *i*, respectively; *A_ji_* is the NPP value for image element *j* in year *i*; and *X_ji_*, *Y_ji_* are the latitude and longitude coordinates of the center of image element *j* in year *i*.

#### 4.3.4. Partial Correlation Analysis

Partial correlation analysis can more clearly identify the degree of direct association between two variables after removing the effects of other confounding factors [35]. Thus, we conducted partial correlation analysis to analyze the degree of influence of different climate variables (temperature, precipitation, and solar radiation) on the vegetation NPP on the NSTM from 2000 to 2020 using the following formulae [94]:

(14) $${\;\;\;\;\;\;\;\;\;\;\;\;\;\;\;\;\;\;\;\;\;\;\;\;\;\;\;R}_{xy}=\frac{\sum_{i=1}^{n}\;\left[\left(x_{i}-\bar{x}\right)\left(y_{i}-\bar{y}\right)\right]}{\sqrt{\sum_{i=1}^{n}\;{\left(x_{i}-\bar{x}\right)}^{2}}\sqrt{\sum_{i=1}^{n}\;{\left(y_{i}-\bar{y}\right)}^{2}}}$$

(15) $$R_{y1\cdot 23}=\frac{R_{y1\cdot 2}-R_{y3\cdot 2}R_{13\cdot 2}}{\sqrt{{\left(1-R_{y3\cdot 2}\right)}^{2}}\sqrt{{\left(1-R_{13\cdot 2}\right)}^{2}}}$$

where *R_xy_* denotes the correlation coefficient between vegetation NPP and individual factors, and *R_y_*_1∙23_ is the partial correlation coefficient between NPP (*y*) and Factor 1 after fixing Factors 2 and 3. The significance of partial correlation coefficients was assessed using the *t*-test (*p* < 0.05).

#### 4.3.5. OPGD Model

The GDM is a widely used statistical model for analyzing spatial stratified heterogeneity, and its analytical functions mainly include factor detection, interaction effect detection, risk detection, and ecological detection. The basic principle of GDM involves using the q value to quantitatively evaluate the spatial relationship between the explanatory variable and target variable, where the q value is an indicator for measuring the explanatory power of the factor [95]. The GDM examines the coupling between the target variable and explanatory variable based on their spatially hierarchical heterogeneity, assuming that there is no linear relationship between them. In addition, the GDM can be used to study the interactions between two explanatory variables (such as X1 and X2) and the target variable (Y) without considering any specific form of interaction. In order to improve the accuracy of the model for analyzing the spatial interactions between explanatory variables and target variables, the effect of the spatial analysis scale on the research results should first be considered. Moreover, continuous spatial variables must be discretized according to their physical properties or distribution characteristics, and different discretized variables must be compared at the optimal spatial scale, thereby improving the identification and quantification of the relationships between the explanatory variables and target variables, and thus optimizing the overall performance of the model.

**Figure 16:**
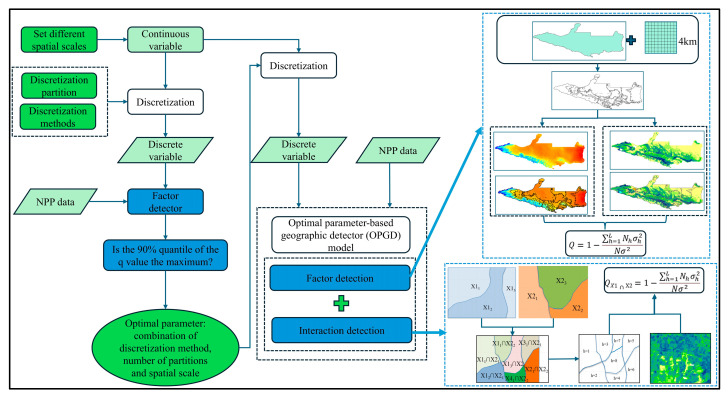
Schematic diagram illustrating the OPGD model.

Song et al. [53] proposed the OPGD model to address the challenges described above. The spatial scale and spatial data discretization method are generally determined based on subjective judgment for the traditional GDM, and the impact of the spatial analysis scale on the research results tends to be ignored. The OPGD model based on the optimal parameters can identify the optimal combination of the spatial scale, spatial data discretization method, and number of partitions to effectively avoid the influence of subjective judgment, thereby improving the scientific reliability and stability of the spatial analysis results [96]. The first step in the OPGD model involves determining the optimal spatial scale and discretization method. According to the size of the study area and previous research results [97], we considered eight spatial scales (3 km, 4 km, 5 km, 6 km, 7 km, 8 km, 9 km, and 10 km), and selected the optimal spatial scale based on the 90% quantile of the explanatory power for each factor at different spatial scales. The discretization method for continuous variables was determined based on the optimal spatial scale. In particular, we determined the optimal discretization method through the factor detection effect (*q* value) of the maximum discretized variable on NPP. The *q* value reflects the strength of the correlation between the discretized variable and NPP, where a larger *q* value indicates that the explanatory power is greater for the discretization method based on the detection results. Finally, we selected the analysis scale and discretization method that could fully analyze the mechanisms of action for the driving factors. Five commonly used discretization methods are the equal interval method, natural break point method, quantile method, geometric interval method, and standard deviation method. Next, we considered different numbers of partitions (set to 3–8) for each discretization method and finally selected the optimal combination of the spatial scale, discretization method, and number of partitions. The second step involved entering the discretized independent variables into the OPGD model at the optimal spatial scale, calculating the *q* value for each factor, conducting factor detection, and evaluating its impact on NPP. The third step involved calculating the *q* value for the interactions between different factors and analyzing how the interactions between multiple explanatory variables affected the target variable. In this study, we constructed the OPGD model with the GD package in the R language environment, mainly using the parameter optimization module, factor detection module, and interaction detection module. This package provides detailed output results and significantly improves the computational efficiency. Based on the results obtained in previous studies [65] and the actual situation in the study area, we selected 10 factors (soil moisture, land use, precipitation, temperature, solar radiation, elevation, GDP, population density, slope, and aspect) from three dimensions consisting of human activities, climate conditions, and natural environment to explore the impact mechanism of each factor on NPP in the study area, the OPGD model construction process is shown in Figure 16. The geographic detector was calculated using the following formula:

(16) $$q=1-\frac{1}{N\sigma^{2}}\sum_{n=1}^{L}\;N_{h}\sigma_{h}^{2}$$

where *q* is the explanatory power of the factor with a range of 0–1, and a larger value denotes the greater explanatory power of the factor; *L* is the number of factor partitions; *N_h_* is the number of samples in partition *h*; *N* is the total number of samples; and $\sigma^{2}$ and $\sigma_{h}^{2}$ are the regional ERI variance and ERI variance in partition *h*, respectively.

## 5. Conclusions

In the present study, we used the CASA model to estimate the vegetation NPP on the NSTM between 2001 and 2020, as well as assessing spatiotemporal changes and the driving mechanism. Our main conclusions are as follows. From 2001 to 2020, the average NPP on the NSTM increased from 107.33 gC⋅m^−2^⋅yr^−1^ to 156.77 gC⋅m^−2^⋅yr^−1^, with a significant overall upward trend, increasing by 2.47 gC⋅m^−2^ per year and 46.06% year-on-year. The spatial distribution of NPP varied significantly, with high NPP values in the south and low values in the north, and high values in the west and low values in the east, where the increase in the NPP was most obvious in the oasis plain area. Over the past 20 years, climate change and human activities have generally positively affected the changes in NPP in the study area, where the large-scale conversion of grassland and unused land into farmland was the main contributor to the increased NPP in the study area. In addition, the vegetation NPP was positively correlated with meteorological factors, where the correlation with precipitation was the strongest. In the study area, the land use type and soil moisture were the main factors that affected the spatial differentiation of vegetation NPP, and analysis of the interactions between factors generally demonstrated that pairs of factors enhanced the NPP in a nonlinear manner. The power of the interactions between factors for explaining changes in the NPP was generally greater than that of any single factor, and land use type acting on natural environmental conditions significantly affected the NPP of vegetation in the study area.

The results obtained in this study provide a scientific basis for understanding the changes in NPP in arid areas. In addition, we introduced the OPGD model to quantitatively study the power of certain factors for explaining changes in vegetation, as well as determining the optimal combination of the optimal spatial scale, spatial data discretization method, number of partitions, and analyzing the driving mechanism to changes in NPP from the perspective of spatiotemporal heterogeneity. This method can be widely used for studying the driving mechanism to changes in NPP in other regions in the future, thereby providing scientific and technological guidance for the construction of an ecological civilization and sustainable development.

## Figures and Tables

**Table 1:** Comparison of the simulation results of this study with those of other scholars (gC⋅m^−2^⋅yr^−1^).

Period	Area	Farmland	Woodland	Grassland	Reference
2001–2020	This study	349.32	510.09	189.11	—
2001–2022	Xinjiang	404.53	454.40	199.88	[20]
2000–2020	Xinjiang	220–208	391.09–482.25	193.06	[60]
2001–2020	ELB	372.16	366.48	212.54	[61]
2001–2020	MRB	355.55–438.72	307.23–496.28	212.08–274.40	[62]
2001–2014	NWCAR	415.60	511.60	252.20	[63]

Note: ELB, Ebinur Lake Basin; MRB: Manas River Basin; NWCAR: Arid region of Northwest China.

**Table 2:** Data sources and information.

Data Type	Data Source	Spatial Resolutions	Temporal Resolutions
NDVI	https://code.earthengine.google.com/ (accessed on 10 March 2025)	500 m	Sixteen days
Vegetation type data	https://code.earthengine.google.com/ (accessed on 10 March 2025)	500 m	One year
NPP	http://www.geodata.cn/ (accessed on 10 March 2025)	500 m	One year
Temperature	https://www.tpdc.ac.cn (accessed on 18 March 2025)	1 km	One month
Precipitation	https://www.tpdc.ac.cn (accessed on 18 March 2025)	1 km	One month
Solar radiation	https://code.earthengine.google.com/ (accessed on 18 March 2025)	4 km	One month
Land use data	http://www.geodata.cn/ (accessed on 25 March 2025)	30 m	One year
Soil moisture	https://www.tpdc.ac.cn (accessed on 25 March 2025)	1 km	One year
Population Data	http://www.resdc.cn (accessed on 25 March 2025)	1 km	One year
GDP Data	http://www.resdc.cn (accessed on 25 March 2025)	1 km	One year
DEM	https://www.resdc.cn/ (accessed on 25 March 2025)	250 m	—

**Table 3:** Trend significance grading scale.

Slope	Z Value	Changing Trend in NPP
<0	Z < −2.58	Extremely significant reduction (ESR)
<0	−2.58 < Z < −1.96	Significant reduction (SR)
-	−1.96 ≤ Z ≤ 1.96	No significant change (NSC)
>0	1.96 < Z < 2.58	Significant increase (SI)
>0	Z > 2.58	Extremely significant increase (ESI)

## Data Availability

Data are contained within the article.

## Supplementary Materials

The following supporting information can be downloaded at: https://www.mdpi.com/article/10.3390/plants14162499/s1, Figure S1. Spatial changes in land use on the NSTM from 2001 to 2020. Figure S2. Changes in vegetation NPP with elevation and slope.

## References

[1] Cao M., Woodward F.I. (1998). Dynamic responses of terrestrial ecosystem carbon cycling to global climate change. Nature.

[2] Pan Y., Birdsey R.A., Fang J., Houghton R., Kauppi P.E., Kurz W.A., Phillips O.L., Shvidenko A., Lewis S.L., Canadell J.G. (2011). A large and persistent carbon sink in the world’s forests. Science.

[3] Jiao K., Liu Z., Wang W., Yu K., Mcgrath M.J., Xu W. (2024). Carbon cycle responses to climate change across China’s terrestrial ecosystem: Sensitivity and driving process. Sci. Total Environ..

[4] Zhang Y., Zhang C., Wang Z., Chen Y., Gang C., An R., Li J. (2016). Vegetation dynamics and its driving forces from climate change and human activities in the Three-River Source Region, China from 1982 to 2012. Sci. Total Environ..

[5] Liu Y., Yang Y., Wang Q., Du X., Li J., Gang C., Zhou W., Wang Z. (2019). Evaluating the responses of net primary productivity and carbon use efficiency of global grassland to climate variability along an aridity gradient. Sci. Total Environ..

[6] Yue D., Zhou Y., Guo J., Chao Z., Liang G., Zheng X. (2022). Ecosystem service evaluation and optimisation in the Shule River Basin, China. Catena.

[7] Ma Z., Wu J., Yang H., Hong Z., Yang J., Gao L. (2024). Assessment of vegetation net primary productivity variation and influencing factors in the Beijing-Tianjin-Hebei region. J. Environ. Manag..

[8] Ruimy A., Saugier B., Dedieu G. (1994). Methodology for the estimation of terrestrial net primary production from remotely sensed data. J. Geophys. Res. Atmos..

[9] Xu Y., Xiao F., Yu L. (2020). Review of spatio-temporal distribution of net primary productity in forest ecosystem and its responses to climate change in China. Acta Ecol. Sin..

[10] Hou G., Wu S., Long W., Chen C., Zhang Z., Fang Y., Zhang Y., Luo G. (2023). Quantitative analysis of the impact of climate change and oasification on changes in net primary productivity variation in mid-Tianshan Mountains from 2001 to 2020. Ecol. Indic..

[11] Luo G., Feng Y., Zhang B., Cheng W. (2010). Sustainable land-use patterns for arid lands: A case study in the northern slope areas of the Tianshan Mountains. J. Geogr. Sci..

[12] Zhang Q., Luo G., Li L., Zhang M., Lv N., Wang X. (2017). An analysis of oasis evolution based on land use and land cover change: A case study in the Sangong River Basin on the northern slope of the Tianshan Mountains. J. Geogr. Sci..

[13] Li L., Wang S., Chen Y., Zhang H., Zhang J., Xu Y., Wei J. (2023). Climate change in the Eastern Xinjiang of China and its connection to northwestern warm humidification. Atmosphere.

[14] Hu Z., Zhang C., Hu Q., Tian H. (2014). Temperature changes in Central Asia from 1979 to 2011 based on multiple datasets. J. Clim..

[15] Chen Y., Li Z., Fang G., Deng H. (2017). Impact of climate change on water resources in the Tianshan Mountians, Central Asia. Acta Geogr. Sin..

[16] Zou J., Ding J., Welp M., Huang S., Liu B. (2020). Assessing the response of ecosystem water use efficiency to drought during and after drought events across Central Asia. Sensors.

[17] Gower S.T., Kucharik C.J., Norman J.M. (1999). Direct and indirect estimation of leaf area index, fAPAR, and net primary production of terrestrial ecosystems. Remote Sens. Environ..

[18] Scurlock J., Cramer W., Olson R., Parton W., Prince S. (1999). Terrestrial NPP: Toward a consistent data set forglobal model evaluation. Ecol. Appl..

[19] Luo T., Pan Y., Ouyang H., Shi P., Luo J., Yu Z., Lu Q. (2004). Leaf area index and net primary productivity along subtropical to alpine gradients in the Tibetan Plateau. Glob. Ecol. Biogeogr..

[20] He X., Zhang F., Zhou T., Xu Y., Xu Y., Jim C.Y., Johnson B.A., Ma X. (2025). Human activities dominated terrestrial productivity increase over the past 22 years in typical arid and semiarid regions of Xinjiang, China. Catena.

[21] Li C., Sun H., Wu X., Han H. (2019). Dataset of the net primary production on the Qinghai-Tibetan Plateau using a soil water content improved Biome-BGC model. Data Brief.

[22] Pan Y., McGuire A.D., Kicklighter D.W., Melillo J.M. (1996). The importance of climate and soils for estimates of net primary production: A sensitivity analysis with the terrestrial ecosystem model. Glob. Change Biol..

[23] Wang P., Xie D., Zhou Y., E Y., Zhu Q. (2014). Estimation of net primary productivity using a process-based model in Gansu Province, Northwest China. Environ. Earth Sci..

[24] Zaks D.P., Ramankutty N., Barford C.C., Foley J.A. (2007). From Miami to Madison: Investigating the relationship between climate and terrestrial net primary production. Glob. Biogeochem. Cycles.

[25] Pranuthi G., Dubey S.K., Tripathi S. (2012). Comparison of different models for estimation of net primary productivity in India. J. Agrometeorol..

[26] Yu R. (2020). An improved estimation of net primary productivity of grassland in the Qinghai-Tibet region using light use efficiency with vegetation photosynthesis model. Ecol. Model..

[27] Sannigrahi S. (2017). Modeling terrestrial ecosystem productivity of an estuarine ecosystem in the Sundarban Biosphere Region, India using seven ecosystem models. Ecol. Model..

[28] Zhu P., Liu G., He J. (2023). Spatio-temporal variation and impacting factors of NPP from 2001 to 2020 in Sanjiangyuan region, China: A deep neural network-based quantitative estimation approach. Ecol. Inform..

[29] He C., Liu Z., Xu M., Ma Q., Dou Y. (2017). Urban expansion brought stress to food security in China: Evidence from decreased cropland net primary productivity. Sci. Total Environ..

[30] Zhang J., Wang J., Chen Y., Huang S., Liang B. (2024). Spatiotemporal variation and prediction of NPP in Beijing-Tianjin-Hebei region by coupling PLUS and CASA models. Ecol. Inform..

[31] Potter C., Klooster S., Genovese V., Hiatt C. (2013). Forest production predicted from satellite image analysis for the Southeast Asia region. Carbon Balance Manag..

[32] Jay S., Potter C., Crabtree R., Genovese V., Weiss D.J., Kraft M. (2016). Evaluation of modelled net primary production using MODIS and landsat satellite data fusion. Carbon Balance Manag..

[33] Zhu W., Pan Y., Zhang J. (2007). Estimation of net primary productivity of chinese terrestrial vegetation based on remote sensing. Chin. J. Plant Ecol..

[34] Chen J., Shao Z., Huang X., Hu B. (2024). Multi-source data-driven estimation of urban net primary productivity: A case study of Wuhan. Int. J. Appl. Earth Obs. Geoinf..

[35] Mao R., Xing L., Wu Q., Song J., Li Q., Long Y., Shi Y., Huang P., Zhang Q. (2024). Evaluating net primary productivity dynamics and their response to land-use change in the loess plateau after the’Grain for Green’program. J. Environ. Manag..

[36] Lu Z., Chen P., Yang Y., Zhang S., Zhang C., Zhu H. (2023). Exploring quantification and analyzing driving force for spatial and temporal differentiation characteristics of vegetation net primary productivity in Shandong Province, China. Ecol. Indic..

[37] Tobias J.A., Durant S.M., Pettorelli N. (2021). Improving predictions of climate change–land use change interactions. Trends Ecol. Evol..

[38] Chowdhuri I., Pal S.C. (2024). Hydrochemical properties of groundwater and land use and land cover changes impact on agricultural productivity: An empirical observation and integrated framework approaches. J. Geochem. Explor..

[39] Roy P., Pal S.C., Chakrabortty R., Chowdhuri I., Malik S., Das B. (2020). Threats of climate and land use change on future flood susceptibility. J. Clean. Prod..

[40] Pal S.C., Chowdhuri I., Das B., Chakrabortty R., Roy P., Saha A., Shit M. (2022). Threats of climate change and land use patterns enhance the susceptibility of future floods in India. J. Environ. Manag..

[41] Hua Y., Wei Y., Chen L. (2025). The influences of climate factors on net primary productivity of Gannan grassland, the Qinghai-Tibet Plateau, China. Theor. Appl. Climatol..

[42] Xiong Q., Xiao Y., Halmy M.W.A., Dakhil M.A., Liang P., Liu C., Zhang L., Pandey B., Pan K., El Kafraway S.B. (2019). Monitoring the impact of climate change and human activities on grassland vegetation dynamics in the northeastern Qinghai-Tibet Plateau of China during 2000–2015. J. Arid Land.

[43] Tao J., Xie Y., Wang W., Zhu J., Zhang Y., Zhang X. (2022). Elevational Gradient of Climate-Driving Effects on Cropland Ecosystem Net Primary Productivity in Alpine Region of the Southwest China. Remote Sens..

[44] Cai Y., Liu X., Liu K., Zeng L., Pei F., Zhuang H., Wen Y., Wu C., Li B. (2024). Human activities significantly impact China’s net primary production variation from 2001 to 2020. Prog. Phys. Geogr. Earth Environ..

[45] Liang S., Peng S., Chen Y. (2019). Carbon cycles of forest ecosystems in a typical climate transition zone under future climate change: A case study of Shaanxi Province, China. Forests.

[46] Xu Q., Li J., Zhang S., Yuan Q., Ren P. (2024). Spatio-Temporal Changes and Driving Mechanisms of Vegetation Net Primary Productivity in Xinjiang, China from 2001 to 2022. Land.

[47] Li J., Feng Q., Guo Q. (2008). Fractal study of sustainable proportions of natural and artificial oases. Environ. Geol..

[48] Song K., Cheng W., Wang B., Xu H., Wang R., Zhang Y. (2024). Study on the Expansion Potential of Artificial Oases in Xinjiang by Coupling Geomorphic Features and Hierarchical Clustering. Remote Sens..

[49] Liu H., Zhang M., Lin Z., Xu X. (2018). Spatial heterogeneity of the relationship between vegetation dynamics and climate change and their driving forces at multiple time scales in Southwest China. Agric. For. Meteorol..

[50] Hao X., Wang X., Ma J., Chen Y., Luo S. (2023). Spatiotemporal Characteristic Prediction and Driving Factor Analysis of Vegetation Net Primary Productivity in Central China Covering the Period of 2001–2019. Land.

[51] Yin Y., Ma D., Wu S., Dai E., Zhu Z., Myneni R.B. (2017). Nonlinear variations of forest leaf area index over China during 1982–2010 based on EEMD method. Int. J. Biometeorol..

[52] Pan N., Feng X., Fu B., Wang S., Ji F., Pan S. (2018). Increasing global vegetation browning hidden in overall vegetation greening: Insights from time-varying trends. Remote Sens. Environ..

[53] Song Y., Wang J., Ge Y., Xu C. (2020). An optimal parameters-based geographical detector model enhances geographic characteristics of explanatory variables for spatial heterogeneity analysis: Cases with different types of spatial data. GIScience Remote Sens..

[54] Zhang W., Xi M., Liu H., Zheng H. (2023). Low sensitivity of net primary productivity to climatic factors in three karst provinces in southwest China from 1981 to 2019. Ecol. Indic..

[55] Shi S., Zhu L., Luo Z., Qiu H. (2023). Quantitative analysis of the contributions of climatic and anthropogenic factors to the variation in net primary productivity, China. Remote Sens..

[56] Du Y., Li X., He X., Li X., Yang G., Li D., Xu W., Qiao X., Li C., Sui L. (2022). Multi-scenario simulation and trade-off analysis of ecological service value in the Manas River Basin based on land use optimization in China. Int. J. Environ. Res. Public Health.

[57] Dang C., Shao Z., Huang X., Zhuang Q., Cheng G., Qian J. (2023). Climate warming-induced phenology changes dominate vegetation productivity in Northern Hemisphere ecosystems. Ecol. Indic..

[58] Zhao M., Running S.W. (2010). Drought-induced reduction in global terrestrial net primary production from 2000 through 2009. Science.

[59] Jiang X., Shen W., Bai X. (2019). Response of net primary productivity to vegetation restoration in Chinese Loess Plateau during 1986–2015. PLoS ONE.

[60] Yisilayili G., He B., Song Y., Luo X., Yang W., Chen Y. (2025). Simulation of Vegetation NPP in Typical Arid Regions Based on the CASA Model and Quantification of Its Driving Factors. Land.

[61] Luo J., Halike A., Duan Y., Yao K., Yao L., Tang H., Tuheti B. (2025). Spatiotemporal dynamics and driving factors of net primary productivity in the Ebinur Lake Basin. Acta Ecol. Sin..

[62] He Y., Ma S., Zhang L., Zhang Y., He L. (2023). Spatio-temporal change of net primary productivity and the evaluatation of the importance of biodiversity maintenance functions in Manas River Basin. Acta Ecol. Sin..

[63] Jiao W., Chen Y., Li Z. (2017). Remote sensing estimation and the reasons for temporal-spatial differences of vegetation net primary productivity in arid region of Northwest China. Chin. J. Ecol..

[64] Li W., Zhou J., Xu Z., Liang Y., Shi J., Zhao X. (2023). Climate impact greater on vegetation NPP but human enhance benefits after the Grain for Green Program in Loess Plateau. Ecol. Indic..

[65] Ge W., Deng L., Wang F., Han J. (2021). Quantifying the contributions of human activities and climate change to vegetation net primary productivity dynamics in China from 2001 to 2016. Sci. Total Environ..

[66] Liao X., Fang C., Shu T. (2022). Multifaceted land use change and varied responses of ecological carrying capacity: A case study of Chongqing, China. Appl. Geogr..

[67] Jiang S., Chen X., Smettem K., Wang T. (2021). Climate and land use influences on changing spatiotemporal patterns of mountain vegetation cover in southwest China. Ecol. Indic..

[68] Zhai Y., Wang Y., Hao L., Qi W. (2025). Medium-and long-term independent contributions of climate change, management measures and land conversion to vegetation dynamics and inspiration for ecological restoration in Inner Mongolia, China. Ecol. Eng..

[69] Wu K., Zhou C., Zhang Y., Xu Y. (2022). Long-term spatiotemporal variation of net primary productivity and its correlation with the urbanization: A case study in Hubei Province, China. Front. Environ. Sci..

[70] Gao J., Liu M., Wang X. (2024). Unveiling the Impact of Urbanization on Net Primary Productivity: Insights from the Yangtze River Delta Urban Agglomeration. Land.

[71] Guang Y., Dong C., Xinlin H., Aihua L., Mingjie Y., Xiaolong L. (2017). Land use change characteristics affected by water saving practices in Manas River Basin, China using Landsat satellite images. Int. J. Agric. Biol. Eng..

[72] Ye C., Sun J., Liu M., Xiong J., Zong N., Hu J., Huang Y., Duan X., Tsunekawa A. (2020). Concurrent and lagged effects of extreme drought induce net reduction in vegetation carbon uptake on Tibetan Plateau. Remote Sens..

[73] Niu S., Wu M., Han Y., Xia J., Li L., Wan S. (2008). Water-mediated responses of ecosystem carbon fluxes to climatic change in a temperate steppe. New Phytol..

[74] Wang J., Sun H., Xiong J., He D., Cheng W., Ye C., Yong Z., Huang X. (2021). Dynamics and drivers of vegetation phenology in three-river headwaters region based on the Google Earth engine. Remote Sens..

[75] Guo D., Song X., Hu R., Cai S., Zhu X., Hao Y. (2021). Grassland type-dependent spatiotemporal characteristics of productivity in Inner Mongolia and its response to climate factors. Sci. Total Environ..

[76] Hao L., Wang S., Cui X., Zhai Y. (2021). Spatiotemporal dynamics of vegetation net primary productivity and its response to climate change in inner Mongolia from 2002 to 2019. Sustainability.

[77] Luo M., Liu T., Meng F., Duan Y., Bao A., Xing W., Feng X., De Maeyer P., Frankl A. (2019). Identifying climate change impacts on water resources in Xinjiang, China. Sci. Total Environ..

[78] Wang Y., Gu X., Yang G., Yao J., Liao N. (2021). Impacts of climate change and human activities on water resources in the Ebinur Lake Basin, Northwest China. J. Arid Land.

[79] Chen Y., Rusuli Y., Wusiman A. (2024). Analysis of spatial and temporal variation in grassland vegetation cover in Xinjiang section ofTianshan Mountains and the driving factors from 2001 to 2020. Chin. J. Plant Ecol..

[80] Walsh S.J., Crawford T.W., Welsh W.F., Crews-Meyer K.A. (2001). A multiscale analysis of LULC and NDVI variation in Nang Rong district, northeast Thailand. Agric. Ecosyst. Environ..

[81] Li Z., Lu Y., Wang Y., Liu J. (2022). The Spatio-temporal evolution of the soil conservation function of ecosystems in the north–south transition zone in China: A case study of the Qinling-Daba Mountains. Sustainability.

[82] Li C., Wang Y., Wu X., Cao H., Li W., Wu T. (2021). Reducing human activity promotes environmental restoration in arid and semi-arid regions: A case study in Northwest China. Sci. Total Environ..

[83] Li Z., Pan J. (2018). Spatiotemporal changes in vegetation net primary productivity in the arid region of Northwest China, 2001 to 2012. Front. Earth Sci..

[84] Wang D., Qin W., Jia G., Shan Z., Hao M. (2022). Assessing the effects of climate variability and vegetation conversion on variations of net primary productivity in the mountainous area of North China. For. Ecol. Manag..

[85] Sannigrahi S., Pilla F., Basu B., Basu A.S., Sarkar K., Chakraborti S., Joshi P.K., Zhang Q., Wang Y., Bhatt S. (2020). Examining the effects of forest fire on terrestrial carbon emission and ecosystem production in India using remote sensing approaches. Sci. Total Environ..

[86] Meyer G., Black T.A., Jassal R.S., Nesic Z., Coops N.C., Christen A., Fredeen A.L., Spittlehouse D.L., Grant N.J., Foord V.N. (2018). Simulation of net ecosystem productivity of a lodgepole pine forest after mountain pine beetle attack using a modified version of 3-PG. For. Ecol. Manag..

[87] Geng Y., Hisoriev H., Wang G., Ma X., Fan L., Mekhrovar O., Abdullo M., Li J., Li Y. (2025). Time-Lag of Seasonal Effects of Extreme Climate Events on Grassland Productivity Across an Altitudinal Gradient in Tajikistan. Plants.

[88] Du L., Tang L., Zheng X., Li Y. (2024). A global analysis of plant nutrient limitation affected by atmospheric nitrogen and phosphorous deposition. Front. Plant Sci..

[89] Yan J., Zhang D., Liu J., Zhou G. (2014). Interactions between CO_2_ enhancement and N addition on net primary productivity and water-use efficiency in a mesocosm with multiple subtropical tree species. Glob. Change Biol..

[90] Cao Y., Zhang J., Zhang Z., Tang H., Liu L., Liu X., Ma R., Zhang M., Zhang X. (2025). The difference in ecological environmental quality impact factors between human activity zone and non-human activity zone in arid regions: A case study of the northern slope of the Tianshan Mountains. Ecol. Indic..

[91] Yang J., Huang X. (2021). 30 m annual land cover and its dynamics in China from 1990 to 2019. Earth Syst. Sci. Data Discuss..

[92] Chen Z., Chen J., Xu G., Sha Z., Yin J., Li Z. (2023). Estimation and climate impact analysis of terrestrial vegetation net primary productivity in China from 2001 to 2020. Land.

[93] Guo B., Yang F., Fan Y., Zang W. (2023). The dominant driving factors of rocky desertification and their variations in typical mountainous karst areas of Southwest China in the context of global change. Catena.

[94] Shi Y., Song J., Zhang J., Huang P., Sun H., Wu Q., Cheng L., Zhang J., Xing L., Lyu S. (2023). Hydrological response to climate change and human activities in the Bahe River, China. J. Hydrol..

[95] Jiang R., Wu P., Song Y., Wu C., Wang P., Zhong Y. (2022). Factors influencing the adoption of renewable energy in the US residential sector: An optimal parameters-based geographical detector approach. Renew. Energy.

[96] Luo P., Song Y., Wu P. (2021). Spatial disparities in trade-offs: Economic and environmental impacts of road infrastructure on continental level. GIScience Remote Sens..

[97] Li M., Wu D., He H., Yu H., Zhao L., Liu C., Hu Z., Li Q. (2025). Spatio-temporal Evolution and Driving Factors of Carbon Storage in the Yellow River Basin from 1990 to 2020. Ecol. Environ. Sci..

