# Supplementary material for: Spatiotemporal Variation Characteristics and Driving Mechanisms of Net Primary Productivity of Vegetation on Northern Slope of Tianshan Mountains Based on CASA Model, China
Source: Plants (Basel). 2025 Aug 12;14(16):2499. doi: 10.3390/plants14162499 (PMC12389615; doi:10.3390/plants14162499)
Supplement: Supplementary file 1 [file plants-14-02499-s001.zip › plants-3760575-supplementary.pdf]

plants

Article

Spatiotemporal variation characteristics and driving mechanisms of net primary productivity of vegetation on the northern slope of Tianshan Mountains based on CASA model, China

Yongjun Du [1,2], Xiaolong Li [1,2], Xinlin He [1,2*], Quanli Zong [1,3*], Guang Yang [1,2] and Fuchu Zhang [1,2]

[1] College of Water Conservancy & Architectural Engineering, Shihezi University, Shihezi 832000, China; du_yongjun@stu.shzu.edu.cn(Y.D.); lixiaolong409@shzu.edu.cn(X.L.); yangguang@shzu.edu.cn (G.Y.); zfc@stu.shzu.edu.cn (F.Z.);
[2] Key Laboratory of Cold and Arid Regions Eco-Hydraulic Engineering of Xinjiang Production & Construction Corps, Shihezi 832000, China;
[3] College of Resources and Environment, Qingdao Agricultural University, Qingdao, 266109, China;
* Correspondence: hexinlin@shzu.edu.cn (X.H.); zongql@qau.edu.cn (Q.Z.);
Tel.: +86-0993-2057229 (X.H.); +86-0532-58957460 (Q.Z.)

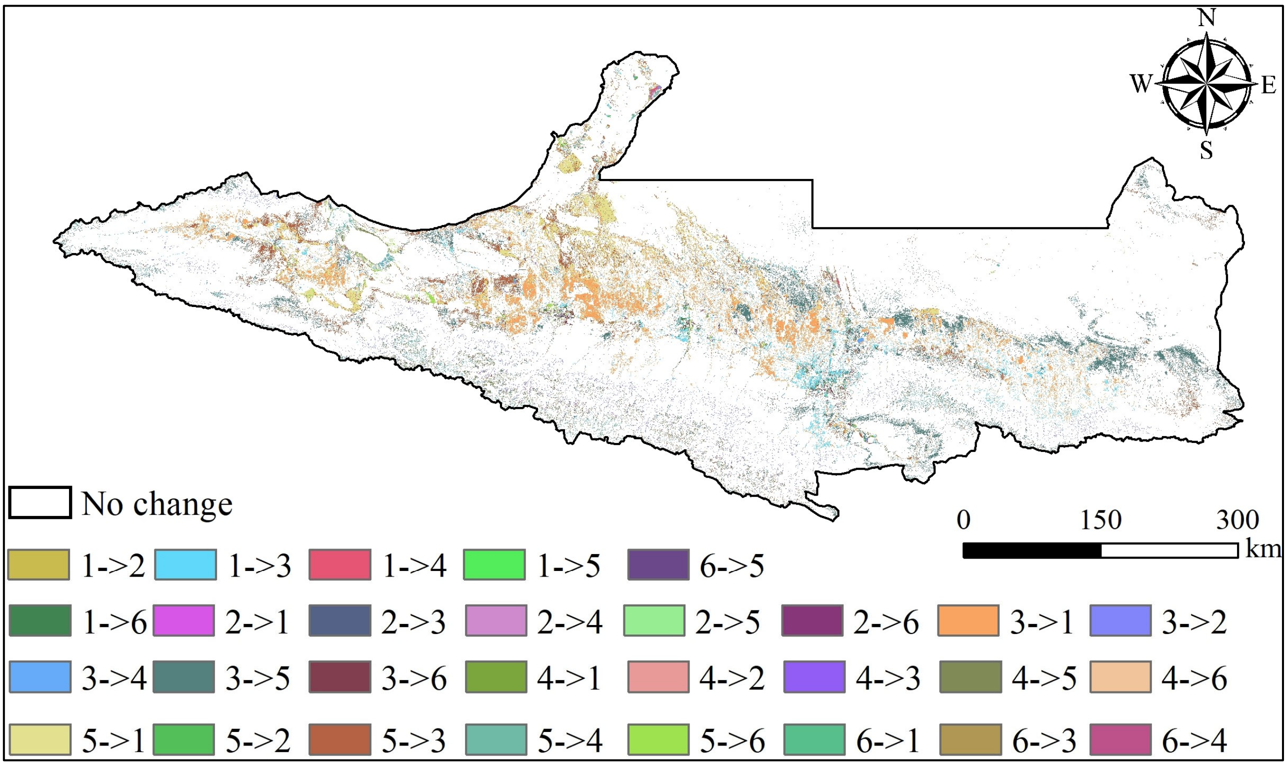

Figure S1 Spatial changes in land use on the NSTM from 2001 to 2020. (1, farmland; 2, woodland; 3, grassland; 4, water body; 5, unused land; 6, construction land.)

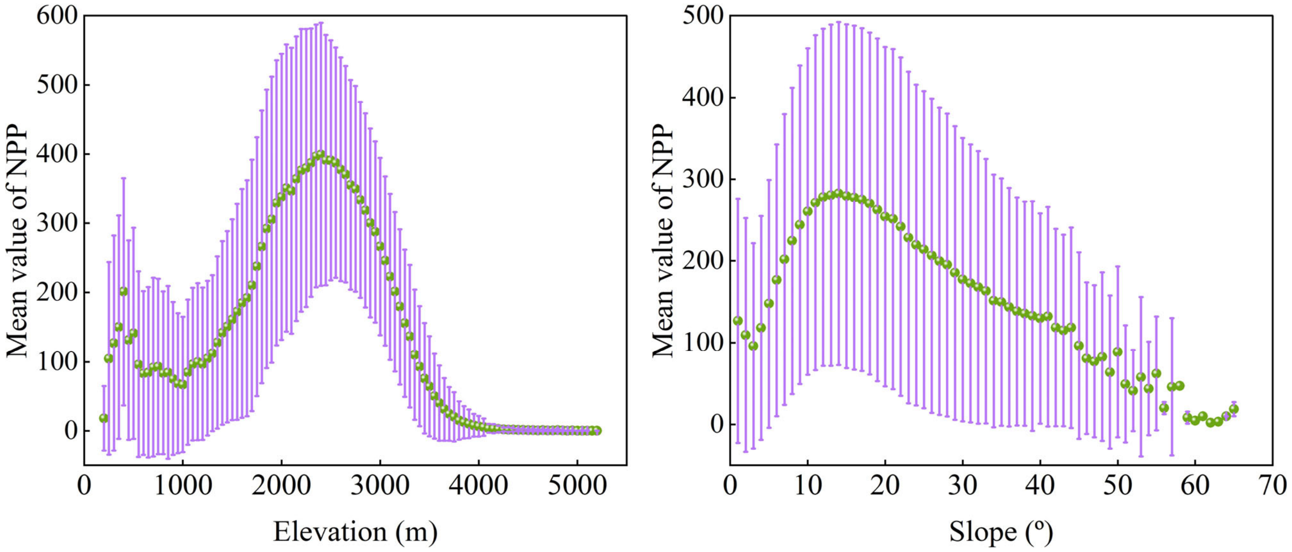

Figure S2 Changes in vegetation NPP with elevation and slope.
